# Comprehensive review of post-treatment imaging in head and neck cancers: from expected to unexpected and beyond

**DOI:** 10.1093/bjr/tqae207

**Published:** 2024-10-11

**Authors:** Nivedita Chakrabarty, Abhishek Mahajan, Archi Agrawal, Kumar Prabhash, Anil K D’Cruz

**Affiliations:** Department of Radiodiagnosis, Advanced Centre for Treatment, Research and Education in Cancer (ACTREC), Tata Memorial Centre, Homi Bhabha National Institute (HBNI), Mumbai 400 012, Maharashtra, India; Faculty of Health and Life Sciences, University of Liverpool, Liverpool, Liverpool L69 3BX, United Kingdom; Department of Imaging, The Clatterbridge Cancer Centre NHS Foundation Trust, Liverpool L7 8YA, United Kingdom; Department of Nuclear Medicine and Molecular Imaging, Tata Memorial Hospital, Homi Bhabha National Institute (HBNI), Mumbai 400 012, Maharashtra, India; Department of Medical Oncology, Tata Memorial Hospital, Tata Memorial Centre, Homi Bhabha National Institute (HBNI), Mumbai 400 012, Maharashtra, India; Director, Department of Oncology, Apollo Hospitals, Navi Mumbai, Maharashtra 400614, India

**Keywords:** post-treatment imaging in head and neck cancers, imaging appearances-recommendations, expected change-complication-recurrence, NI-RADS

## Abstract

Head and neck cancer management requires multidisciplinary approach in which radical surgery with or without flap reconstructions and neck dissection, along with radiotherapy (RT)/chemoradiotherapy (CRT) serve as the key components. Neoadjuvant chemotherapy and immunotherapy are used in selected cases based on the institutional preference. Knowledge of expected post-treatment changes on imaging is essential to differentiate it from recurrence. In addition, awareness of various post-treatment complications is imperative for their early detection on imaging. Distorted anatomy after treatment poses diagnostic challenge, hence, proper choice of imaging modality and appropriate timing of scan is pertinent for accurate post-treatment evaluation. In this article, we have comprehensively reviewed expected post-treatment appearances and complications on imaging. We have discussed imaging appearances of recurrences at the primary and lymphnodal sites and discussed documentation of findings using Neck Imaging Reporting and Data Systems (NI-RADS). We have also delved into the patterns of recurrence in human papillomavirus (HPV) positive HNSCC. Furthermore, we have provided flowcharts and discussed recommendations on the site-specific and treatment-related imaging modalities to be used along with their appropriate timing, for adequate evaluation of HNSCC after treatment. In addition, we have also touched upon the role of advanced imaging techniques for post-treatment HNSCC evaluation.

## Introduction

Lip and oral cavity cancer ranks highest in cancer incidence amongst head and neck squamous cell carcinomas (HNSCC), with 2% new cases worldwide as per GLOBOCAN 2020 data, followed by laryngeal cancer with 0.96% new cases.^1^ Tobacco and alcohol consumption are responsible for approximately 50%-60% of HNSCC.^2^^,^^3^ A strong association of human papillomavirus (HPV) with oropharyngeal carcinoma (OPC), and Ebstein-Barr virus with nasopharyngeal cancers is known.^2^^,^^4^ About 50%-60% of treated HNSCC patients show locoregional recurrence within 2 years and 20%-30% of them develop distant metastasis.^5^

HNSCC management requires multidisciplinary approach with surgery and radiotherapy (RT)/chemoradiotherapy (CRT) being key components and neoadjuvant chemotherapy (NACT) having a selected role based on the institutional preference.^2^^,^^6–10^ Checkmate 141 phase III trial, Keynote-040 and Keynote-048 randomized phase III trials have established the role of immunotherapy for recurrent and metastatic HNSCC, and currently the role of neoadjuvant immunotherapy is being explored.^11^ Altered anatomy after radical surgery with or without flap reconstruction and neck dissection, post-RT/CRT/NACT/immunotherapy expected changes, recurrence, and post-operative or post-RT complications, all pose diagnostic challenges to the radiologist evaluating post-treatment scan.

In this article, we have extensively reviewed post-surgery and post-RT expected changes and complications on imaging, along with post-RT/chemotherapy/immunotherapy response evaluation, recurrence and metastasis detection in HNSCC, and discussed post-treatment assessment using Hopkins criteria and Neck Imaging Reporting and Data Systems (NI-RADS). We have also discussed the current national comprehensive cancer network (NCCN) and European Society for Medical Oncology (ESMO) guidelines on post-treatment HNSCC imaging. Furthermore, we have formulated a flow chart on site-specific and treatment-related imaging modalities to be used along with their appropriate timing for adequate evaluation of HNSCC after treatment, and also provided a summary of imaging recommendations at the end, which can be quickly referred to by radiologists and clinicians to decide upon further line of management. In this article, we have also discussed patterns of recurrence in HPV positive HNSCC and enumerated role of advanced imaging techniques in post-treatment HNSCC evaluation.

## Post-treatment imaging in HNSCC: expected changes and complications

Knowledge of expected/biologic changes on imaging after radical surgery with or without flap reconstructions and neck dissection, RT/CRT, and immunotherapy, is essential for differentiation from recurrence, and early detection of complications.

### Post-surgical evaluation

**Figure 1.**
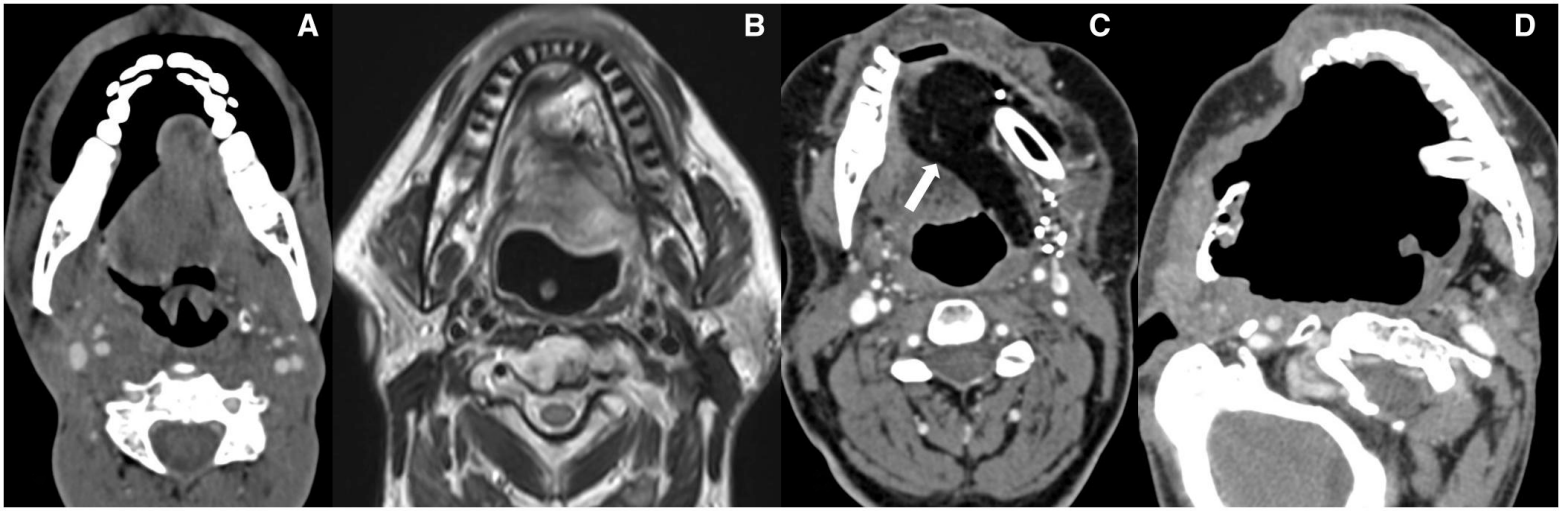
(A) CECT shows post right hemiglossectomy with preserved base of tongue. (B) T2WI shows subtotal hemiglossectomy with removal of mobile tongue on both sides of midline with preserved base of tongue. (C) CECT shows left near total glossectomy and flap reconstruction (arrow) with removal of ipsilateral and contralateral genioglossus and ipsilateral base of tongue. (D) CECT shows total glossectomy with removal of entire tongue.

**Figure 2.**
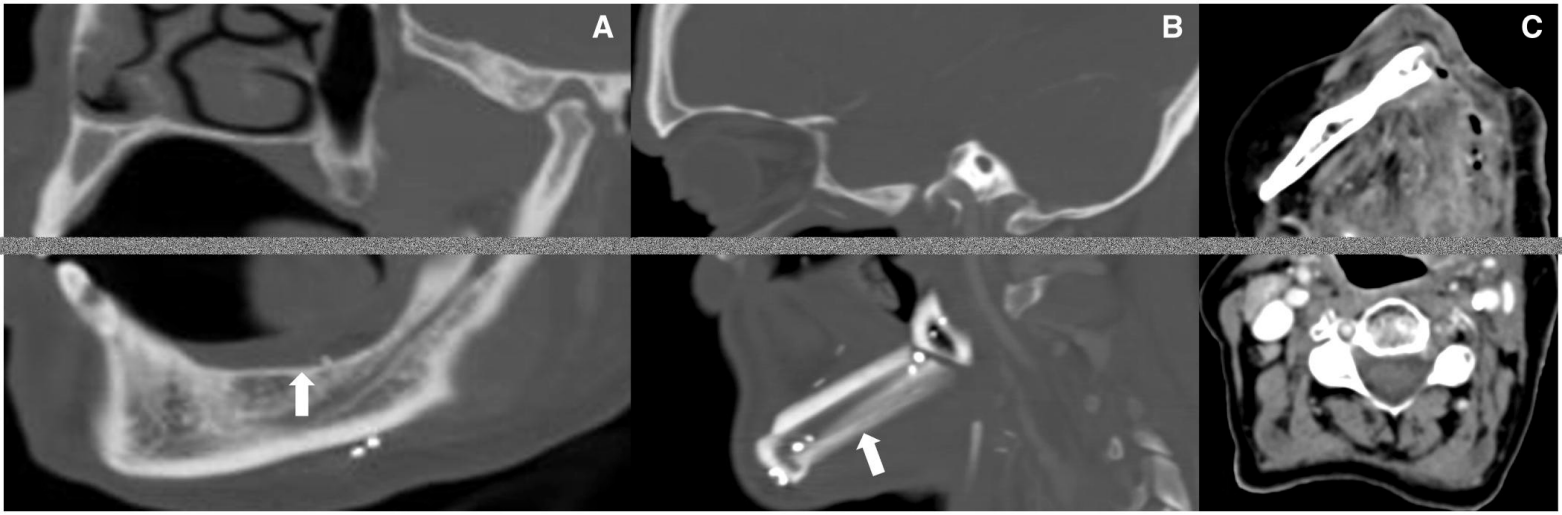
(A) CT with bone algorithm shows post-marginal mandibulectomy status with removal of a rim of mandible including cortex and preserved mandibular continuity and medullary cavity. (B) CT with bone algorithm shows post-segmental mandibulectomy status with removal of a segment of mandible resulting in discontinuity which has been filled with fibular graft (arrow) here (sutures are seen). (C) CECT shows post left hemimandibulectomy status with resection of one half of mandible from midline. In addition, recurrence is seen at the post-operative site.

**Figure 3.**
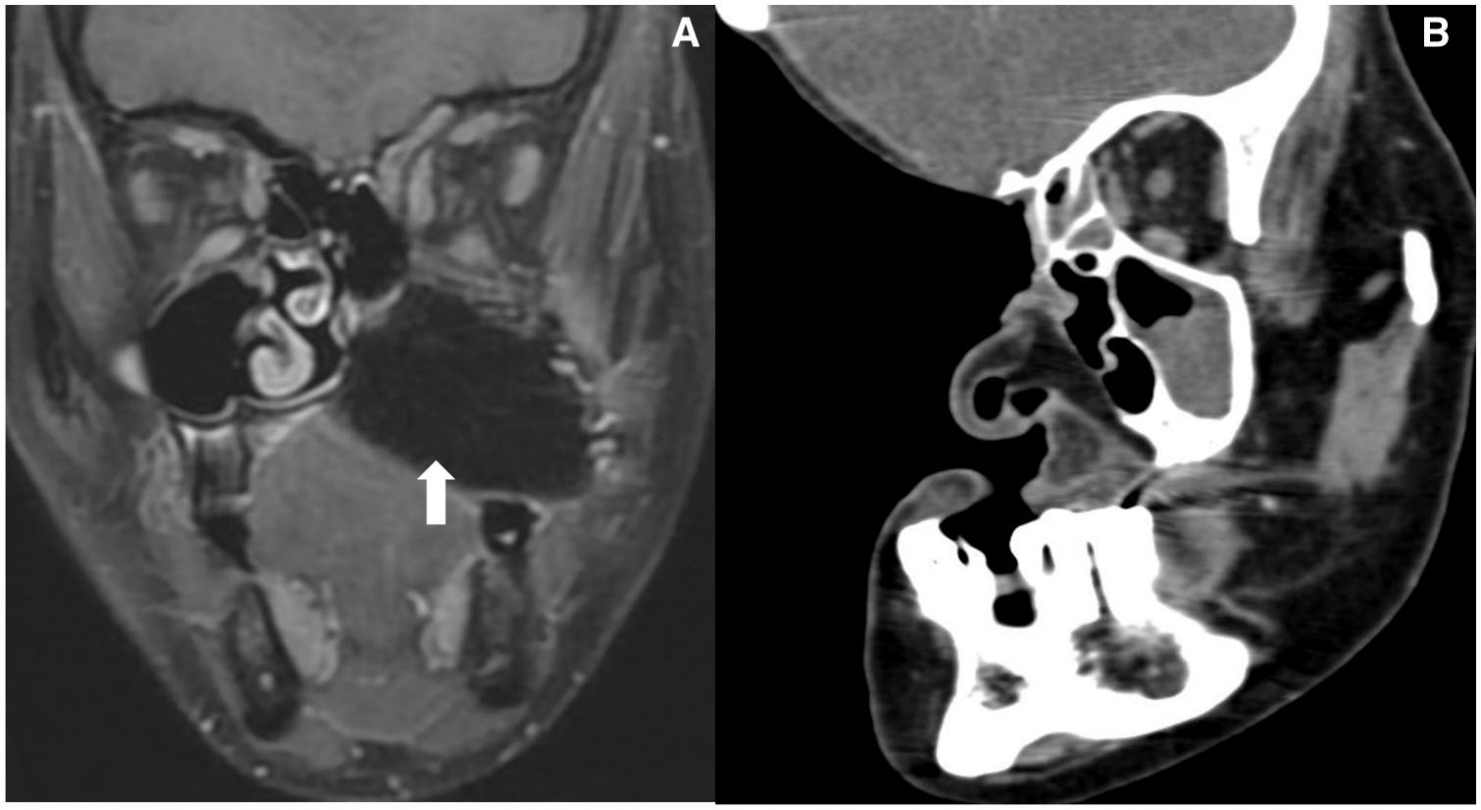
(A) CEMRI shows left subtotal maxillectomy with free flap (arrow) with preserved orbit and zygoma in a case of sinonasal nuclear protein in testis (NUT) carcinoma. (B) CECT shows post-total right maxillectomy with right orbital exenteration of right maxillary adenoid cystic carcinoma who presented with orbital recurrence.

**Figure 4.**
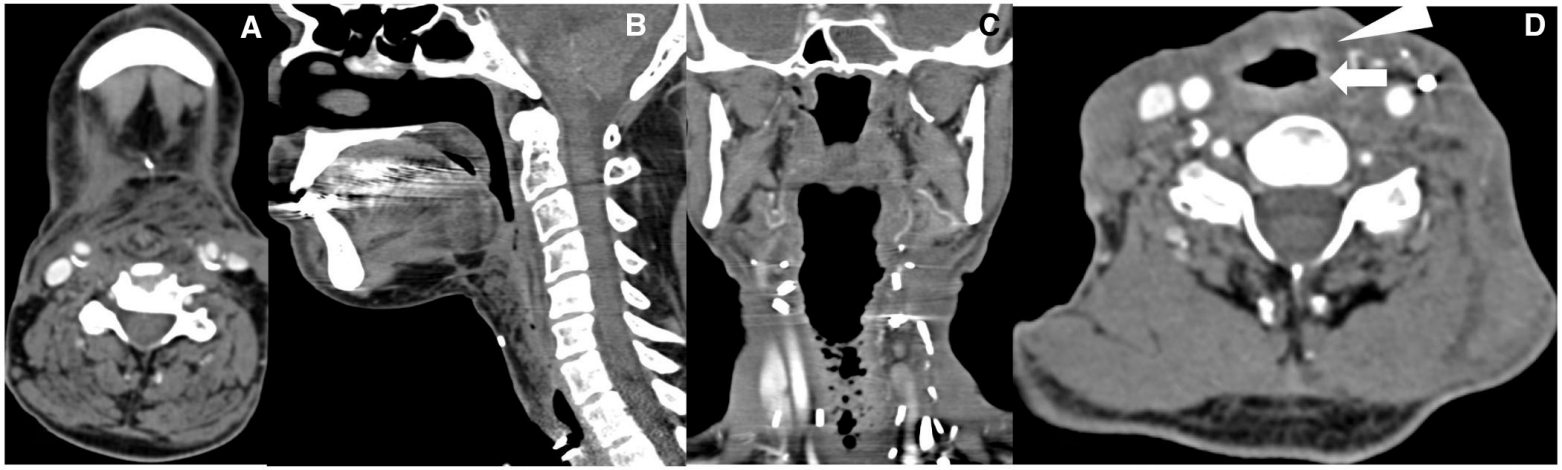
Axial (A) and sagittal (B) images of post-total laryngectomy case of carcinoma subglottis shows complete removal of larynx. In coronal (C) and axial (D) CECT images of post-total laryngopharyngectomy, total thyroidectomy case of carcinoma glottis with free jejunal flap reconstruction, arrow shows hyperdense mucosa and arrowhead shows hypodense submucosa of the neopharynx.

**Figure 5.**
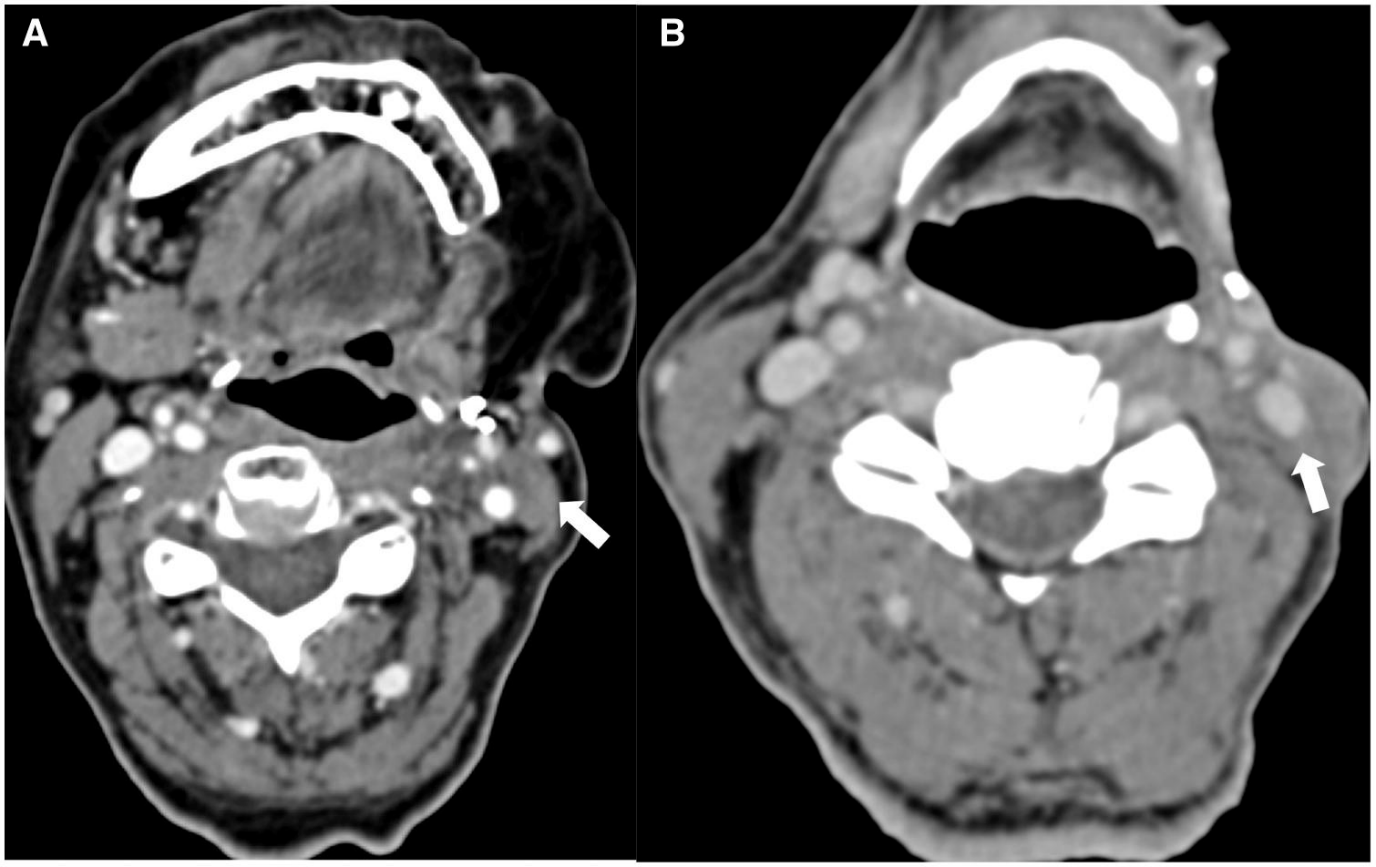
(A) CECT in an operated case of left buccal mucosa carcinoma with left modified neck dissection and flap reconstruction shows preserved left sternocleidomastoid muscle (arrow) and resected left IJV and left submandibular gland. (B) CECT in an operated case of carcinoma left buccal mucosa with left modified neck dissection shows preserved left IJV and expected post-operative soft tissue around left IJV.

Surgery is performed either for curative intent in early stage cancer (T1-T2 N0 [stage I and II] oral cavity, laryngeal, p16-negative oropharyngeal carcinoma or T1-T2N0 p16-positive oropharyngeal cancer as per the American Joint Committee on Cancer [AJCC]/International Union against Cancer [UICC] Tumour Node Metastasis [TNM] 8th edition), for local control in locally advanced HNC (T3/T4 oral cavity and T4 larynx carcinomas), for airway conservation, symptom amelioration, or in selected cases of advanced stage depending upon response to other first line therapies.^2^^,^^6^^,^^12^ Advanced (T4) hypopharyngeal carcinomas with laryngeal cartilage invasion or non-functional larynx also warrant surgical treatment.^2^ Salvage surgery, that is, surgery performed as a last resort after failed initial organ preserving treatment with concurrent CRT (CCRT)/RT, has overall high complication risk of approximately 67% and a locoregional recurrence (LRR) rate of 60% for oral cavity SCC, posing a significant challenge to the radiologists should the need for imaging arises after such a surgery.^13^^,^^14^Table 1 and Figures 1-5 show various types of HNSCC surgeries.^2^^,^^13^^,^^15–27^ Contrast-enhanced CT (CECT) and contrast-enhanced MRI (CEMRI) are the predominant imaging modalities for post-treatment evaluation.

**Table 1.** Types of head and neck squamous cell carcinoma (HNSCC) surgeries.

Minimally invasive surgeries^2^^,^^13^^,^^15^^,^^16^ Lower incidence of post-operative complications due to less morbid surgeries	
Transoral robotic surgery (TORS) for base of tongue and supraglottic cancers and for salvage surgery of early stage recurrent oropharyngeal squamous cell carcinoma	Post-TORS on imaging there is asymmetry of tonsils, with small sized tonsil on the operative side. It is important to know this so as not to mistake larger appearing normal side tonsil to be diseased
Transoral laser microsurgery (TLM) for early glottic cancer and endoscopic resection of sinonasal tumours	On imaging, focal defect is seen at the site of laser surgery which may enhance in early post-operative phase and should not to mistaken for residual disease
Type of glossectomy^17^ Imaging (CT/MRI/fluorodeoxyglucose [FDG]) in the post-operative phase may show mucosal enhancement up to 4 weeks in mucosectomy. In partial to near-total glossectomies with or without flap reconstruction, mucosal enhancement may be seen up to 4-6 weeks. Additional findings pertaining to flap reconstructions may be seen as described in the section on flaps	
Type I glossectomy (mucosectomy) For superficial non-biopsy proven suspicious lesions limited to tongue epithelium	Mucosa, submucosa and a thin layer of intrinsic muscle are removed
Type II glossectomy (partial glossectomy) For lesions involving submucosa and superficial portion of intrinsic muscles	Lesion along with a safety margin of 1.5 cm are removed which includes adjacent normal mucosa, submucosa, and the intrinsic muscles up to the surface of the extrinsic muscles. Defect 4-6 weeks after surgery
Type IIIa glossectomy (hemiglossectomy) For lesions involving intrinsic and minimal extrinsic muscles or DOI > 10 mm restricted to ipsilateral tongue	Mucosa, submucosa, and intrinsic and extrinsic muscles ipsilateral to the lesion are removed with preserved base of tongue. Tip of the tongue may or may not be removed
Type IIIb glossectomy (compartmental hemiglossectomy) For large extent involvement of intrinsic and extrinsic muscles of ipsilateral tongue	Mucosa, submucosa, intrinsic and extrinsic muscles ipsilateral to the lesion, genioglossus, hyoglossus and styloglossus muscles, and the inferior portion of the palatoglossus muscle are removed along with midline raphe
Type IVa glossectomy (subtotal glossectomy) For lesions confined to the mobile tongue but involve contralateral genioglossus muscle as well	Anterior subtotal glossectomy with preservation of both sides of the base of the tongue
Type IVb glossectomy (near-total glossectomy) Large lesions infiltrating ipsilateral tongue base and contralateral genioglossus	Removal of structures in Type IV a glossectomy along with ipsilateral base of the tongue
Type V glossectomy (total glossectomy) Large lesions impairing tongue mobility	Removal of entire mobile tongue along with base of tongue
Type of mandibulectomy^18^ If bone (fibular) graft is used for reconstruction, changes pertaining to the bone graft may be seen as described in the section on flaps. If plates are used for reconstruction, beam hardening artifacts will be seen on post-operative CT unless Iterative Metal Artifact Reduction (iMAR) algorithm is used, and ghost artifacts will be seen on post-operative MRI, hence it is advisable to use Dixon MRI sequence	
Marginal mandibulectomy Performed when no/only superficial cortical erosion is present, preserved height of mandible free of paramandibular soft tissue is more than 1.5 cm	Rim of the mandible is removed which includes a cortical portion and the underlying medullary cavity with preserved mandibular continuity
Segmental mandibulectomy Performed when a segment of mandibular marrow is involved with destruction of mandibular canal, due to either gingivobuccal or retromolar trigone carcinoma or post-radiotherapy destruction	Segment of mandible is resected resulting in discontinuity
Hemimandibulectomy Performed when one half of the mandible is destroyed	Resection of one half of the mandible
Type of maxillectomy^19^ Maxillectomy leads to significant distortion in the imaging anatomy, knowledge of type of surgery is important prior to imaging post-maxillectomy cases	
Limited (Type I)	Removal of one wall
Subtotal (Type II)	Removal of two walls with hard palate
Total	Removal of complete maxilla
Partial maxillectomies can also be classified as follows:	
Infrastructure	Removal of hard palate and upper alveolus below the level of nasal floor
Medial	Removal of medial maxillary wall along with medial 1/3rd of inferior orbital wall and the medial orbital wall
Suprastructure	Removal of all the maxillary walls, barring hard palate and upper alveolus
Subtotal	Removal of all the maxillary walls, barring the orbital floor and the zygomatic buttress
Types of flap reconstructions^20–25^ Extensive surgeries require flap reconstructions to cover a large defect and for function preservation. Early flap imaging is usually not recommended and may be indicated in scenarios with suspected flap necrosis which is usually diagnosed clinically and imaging is reserved for indeterminate cases	
Myocutaneous flaps These are pedicled regional flap with preserved vasculature	Myocutaneous flaps, for example, pectoralis major flap, initially appear as a soft tissue density/intensity structure on CT/MRI respectively, which striations and signal on T1WI representative of a muscle, which eventually undergoes denervation atrophy resulting in volume loss and fatty replacement with fat density/signal intensity on CT/MRI, respectively Sharp boundaries between flap and adjacent normal structures, differentiate it from a recurrent mass Contrast enhancement in myocutaneous flaps range from moderate to intense enhancement in majority, to none
Free flap Here, host tissue (skin, muscle, or bone) remote from the primary tumour site is used to cover the defect and the donor vessel is anastomosed with the recipient vessel and such free flaps also provide protection to vulnerable tissues prior to adjuvant RT	The jejunal free flap, mostly used for reconstruction of the pharyngoesophageal region, may develop reactive lymphadenopathy due to environmental changes post-surgery, and should not be confused with metastatic lymphadenopathy Free flap used for formation of neopharynx shows three layers on CT scan; inner hyperdense enhancing layer of mucosa, middle hypodense layer of submucosa and outer isodense layer of pharyngeal constrictor muscle Within one month following fibular free flap reconstruction, ossification of periosteal vascular pedicle may be evident (in approximately 50% of the patients) seen mostly as linear bone-like structures with cortex and marrow on CT
Type of neck dissection^21^^,^^22^^,^^26^^,^^27^ After neck dissection, fibrosis or scar encircling the carotid sheath is seen as soft tissue attenuating area on CT scan, which on MRI appears as hypointense to isointense on T1 and T2 weighted imaging (WI)	
Radical neck dissection	Removal of levels I-V unilateral neck nodes along with the sternocleidomastoid muscle, internal jugular vein, submandibular gland, and spinal accessory nerve
Modified radical neck dissection	One or more of the following are preserved: spinal accessory nerve, sternocleidomastoid muscle, internal jugular vein, or submandibular gland, and rest of the structures are removed as in radical neck dissection
Selective neck dissection	Selective nodes are removed, for example, levels I-III in supraomohyoid neck dissection, levels II-IV in lateral neck dissection, levels II-V in posterolateral neck dissection, and levels VI and VII in anterior compartment neck dissection
Extended neck dissection	Removal of additional lymph node groups (one or more) and/or non-lymphatic structures beyond the confines of radical neck dissection

#### Imaging of post-operative complications

**Figure 6.**
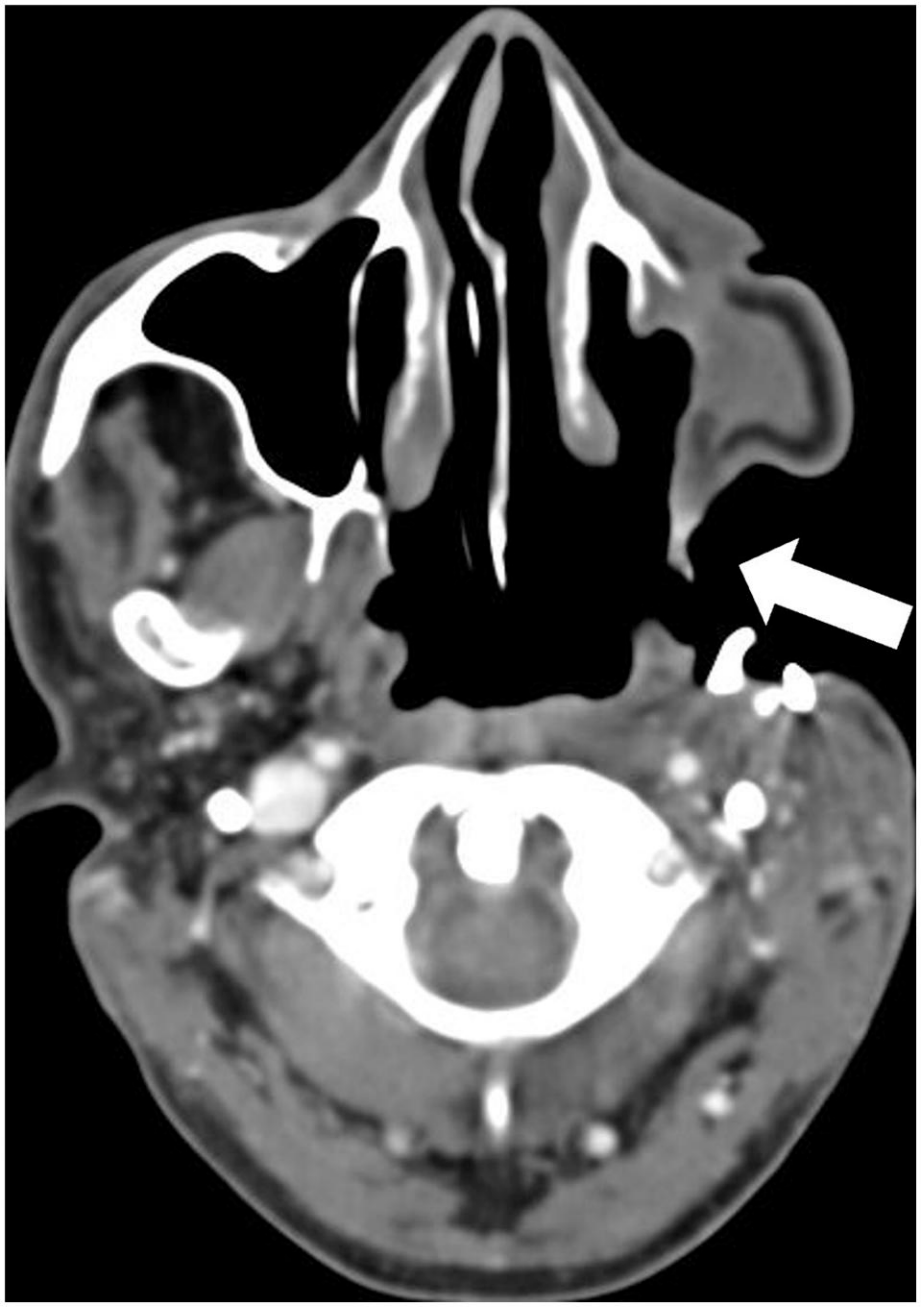
CECT in a left buccal mucosa carcinoma post-segmental composite resection and infratemporal fossa (ITF) clearance shows orocutaneous fistula (arrow).

Post-operative complications occur early and may be categorized as follows: (a) Complications associated with radical surgery with or without flap reconstructions^22^: This includes seroma; which appears as non-enhancing fluid collection, abscess; as a rim enhancing fluid collection showing diffusion restriction on MRI, haematoma; which appears hyperdense on CT and may show T1 hyperintensity and mixed signal on T2 due to blood on MRI, and pharyngocutaneous fistula (Figure 6); seen as a direct visualization of fistulous track communicating between the oral cavity/larynx and cutis. (b) Complications specific to flap reconstructions^22^: This includes flap necrosis due to arterial or venous thrombosis seen as non-opacification of neck vessels on CECT with non-enhancement of flap and loss of flow void on T1 and T2W MRI with filling defect on CEMRI and doppler. Infection is another cause of flap necrosis seen as rim enhancing collections with air locules within the flap/surgical site. Implantable doppler and skin paddle monitor are used to monitor flap perfusion,^22^^,^^23^ venous thrombosis is a common cause of flap failure and may be seen within 3 days of surgery.^22^ Radial forearm and fibular osteocutaneous free flaps are predominantly associated with distal limb ischaemia.^22^ Complications specific to neck dissection: This includes chylous fistula, which may occur in 1%-2% of the patients with level IV neck dissection, and is suspected when collections are located predominantly in left lower neck on imaging.^22^ Pre-operative RT, pre-operative CRT, malnutrition, anaemia, tobacco and alcohol use are risk factors for complications after head and neck surgeries.^22^

Table S1 shows the various types of surgeries and the complications arising thereof.^15^^,^^22^^,^^27–34^

### Evaluation after RT/CRT

RT is a key component of HNSCC treatment, and clinical trials have shown that CCRT reduces risk of death by 19% and overall improved 5 year survival by approximately 8% in comparison to RT alone.^35^ Definitive RT is the mainstay treatment for non-metastatic nasopharyngeal carcinoma.^36^ Definitive CRT may be considered in locally advanced inoperable oral cavity carcinomas.^2^ CCRT is the treatment of choice for locally advanced HNSCC, especially, nasopharyngeal, oropharyngeal, hypopharyngeal, and laryngeal carcinomas.^2^^,^^6^ High-risk factors (mainly for oral cavity carcinomas) for post-operative LRR; pT3-4 on resected specimen according to UICC/AJCC TNM 8th edition, tumour ≤1 mm from the margin (positive margin), tumour between 1 and 5 mm from the margin (close margin), perineural infiltration, lymphovascular involvement, >1 lymph node infiltration, and the presence of extranodal extension, entail adjuvant RT/CCRT.^2^ In general, post-surgery adjuvant RT/CTRT is given when microscopic or gross residual tumour is suspected.^6^

Current RT techniques including intensity modulated RT (IMRT), 3D conformal RT (3DCRT), helical tomotherapy, volumetric modulated arc therapy (VMAT), and proton beam therapy (PBT), spare the critical organs from the radiation field, decrease normal tissue damage, and at the same time achieve primary objective of local tumour control.^6^

#### Imaging of expected/biologic effects after RT/CRT

RT affects tumour microenvironment by causing hypoxia, fibrotic responses, and immune activation, which in turn affects post-RT response to treatment.^37^ CECT and Fluorodeoxy glucose Positron Emission Tomography CECT (FDG-PET/CECT) are the predominant imaging modalities for post-RT/CRT evaluation except for sinonasal and skull base tumours where MRI is the imaging modality of choice.^6^ CEMRI is also reserved for assessment of suspected perineural tumour recurrence, particularly in nasopharyngeal and skull base tumours.^22^

**Figure 7.**
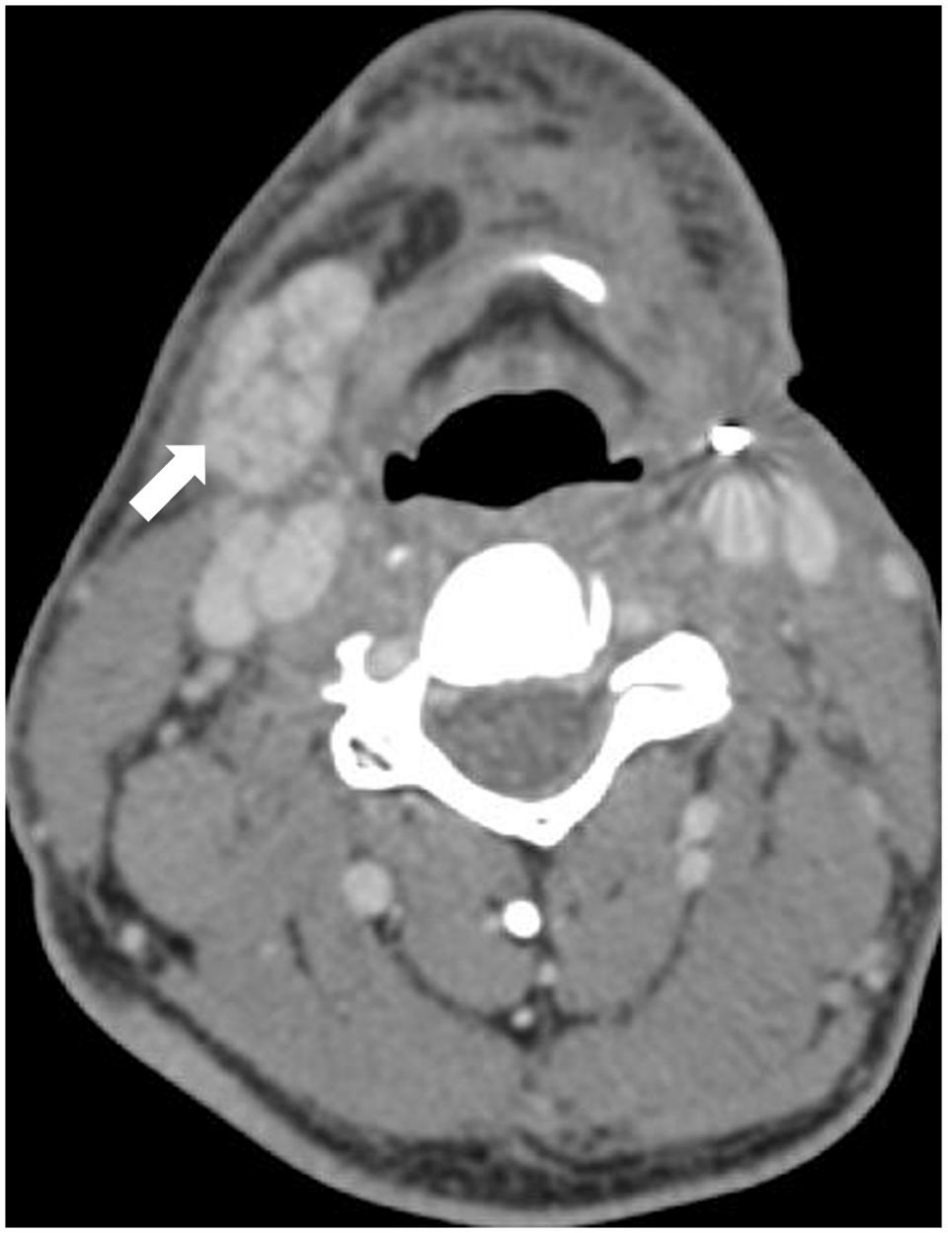
CECT in a post-operative, left modified radical neck dissection (MND) and post-radiotherapy (RT) case of left buccal mucosa carcinoma shows hyperenhancing right submandibular gland (arrow) suggestive of expected early post-RT change.

Expected or biological changes after RT can be divided into early inflammatory and proliferative phases, and delayed tissue remodelling phase.^37^ There is temporal evolution of post-RT biological changes in tissues within the radiation portal and Table 2 describes the early and late imaging findings (CT/MRI) along with underlying pathological changes that occur after RT/CRT.^21^^,^^22^^,^^38^Figure 7 shows expected early post-RT change on CT. On FDG-PET/CECT, early post-RT changes manifest as diffuse, symmetrical, low grade FDG uptake confined to the radiation field.^39^ Groups of muscles within the radiation field may also show increased FDG uptake.^39^

**Table 2.** Expected early and delayed pathologic changes after radiotherapy/chemoradiotherapy along with their imaging correlates.

Time-frame	Pathologic changes	Imaging (CT/MRI) correlates
Early (within 2 weeks to 3 months)	Interstitial oedema Increased permeability within small blood and lymphatic vessels due to detachment of lining endothelial cells Glandular hyperaemia Fatty infiltration of bone marrow Gradual increase in thickening of connective tissues	Subcutaneous fat stranding and reticulations Pharyngeal oedema with increased enhancement of the pharyngeal walls Laryngeal oedema Enlarged submandibular and parotid glands (unilateral or bilateral depending upon the radiation portal) showing intense enhancement Edematous and heterogeneously enhancing thyroid gland (low on T1WI and increased signal on T2WI) Conversion to fatty marrow (seen as hyperintense on T1 and T2WI) strictly within the confines of radiation portal, mainly seen in cervical vertebrae On CT, bones with less marrow space show sclerosis Increase in pre-epiglottic and paralaryngeal fat
Delayed (>90 days)	Fibrosis	Thickening of skin and platysma muscles Thickening of the pharyngeal constrictor muscle Atrophy of salivary glands and thyroid gland

#### Imaging of post-RT/CRT complications

**Figure 8.**
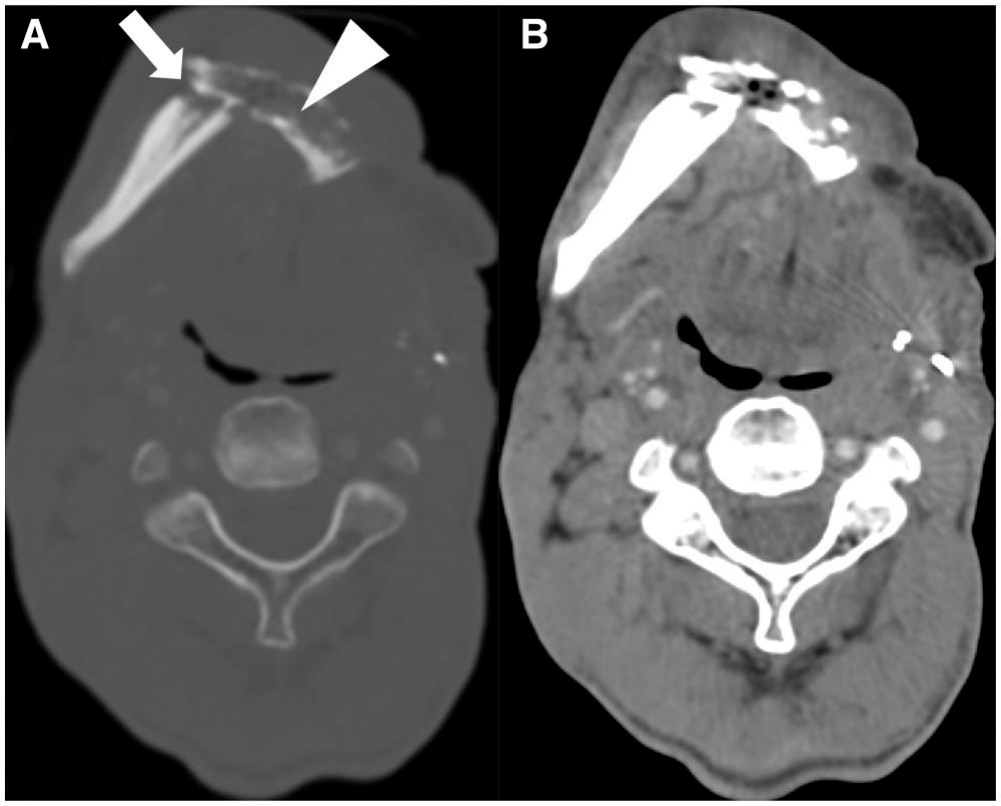
CT with bone algorithm (A) and CECT in soft tissue window (B) in a post-left hemimandibulectomy and post adjuvant chemoradiotherapy (CRT) case of lower gingivobuccal carcinoma shows fractured fragment (arrow) involving body of mandible with multiple lucencies (arrowhead) and without any suspicious enhancing soft tissue, suggestive of osteoradionecrosis (delayed complication of RT).

With the newer RT techniques (IMRT, 3DCRT VMAT, PBT), incidence of post-RT complications have reduced, though not nullified.^6^^,^^22^ Technique and dose of RT, along with the volume of irradiated tissues, tumour location and stage, and poor oral hygiene have bearing on post-RT complications, and alcohol and smoking are known to aggravate RT related complications.^22^ Early post-RT complications like oral mucositis and skin desquamation may get exacerbated due to CCRT and NACT.^22^^,^^40^^,^^41^ Post-operative complications occur more after CRT (46%-100%) than after RT alone (37%-74%).^22^^,^^42^ The various post-RT complications along with their pathologic basis are enumerated in Table 3.^21^^,^^22^^,^^37^^,^^38^^,^^43–50^ and their imaging features are described in Table 4.^21^^,^^22^^,^^38^^,^^42^^,^^43^^,^^51–53^ CT images of osteoradionecrosis (ORN) is shown in Figure 8. It should be noted that osteoradionecrosis may show diffuse inflammation with intense FDG-PET/CECT uptake in the tissues surrounding the lytic and sclerotic bone (Figure S1), however, a focal enhancing soft tissue is absent.

**Table 3.** Post-RT complications along with their underlying pathology.

Post-RT complications	Pathologic basis	Time frame
Mucosal necrosis	Severe vascular congestion and denuded epithelium leads to fibrosis, which in turn leads to impaired microvasculature and lymphatic flow which in turn produces hypoxic, hypovascular tissue with resultant mucosal necrosis and ulceration	6-12 months
Trismus	Lateral pterygoid and temporomandibular joint fibrosis mainly after EBRT for nasopharyngeal carcinoma	12-18 months
Osteoradionecrosis (incidence significantly reduced with the new IMRT techniques)	Non-healing devitalised irradiated bone with persistent fistula for at least 3 months with dose >60 Gy, aggravated by extensive prior surgical resection. Impaired bone formation, increased bone resorption followed by reparative process	1-3 years
Chondroradionecrosis (laryngeal)	Breached perichondrium by tumour exposes underlying irradiated cartilage to infections from airway, resulting in infectious perichondritis, resulting in cartilage necrosis and collapse	1-10 years
Vascular thrombosis and accelerated carotid atherosclerosis	Endothelial proliferation and thickening of intimal layer	4 months to 20 years
Radiation induced lung damage	Lung apices included within the radiation field undergo radiation pneumonitis early and radiation fibrosis later on	Radiation pneumonitis within 1-3 months. Radiation fibrosis 6-12 months
Radiation induced brain necrosis	Medial and inferior temporal lobe necrosis mainly with EBRT technique used in skull base and nasopharyngeal carcinomas due to myelin dysfunction and gliosis	<2 years
Hypoglossal palsy and less commonly vagal neuropathy	Nerve entrapment due to fibrosis	2-10 years
Brachial neuropathy	Fibrosis of the affected roots and trunk	2-4 years
Delayed cord injury	Radiation myelopathy	1-2 years
Radiation induced Herpes Simplex Encephalitis (HSE)	After RT for nasopharyngeal carcinoma Combination of carcinoma, CT, RT, steroids create immunosuppressive state which predisposes to HSE due to disrupted blood brain barrier	2 days to 2 1/2 months
Radiation induced neoplasm: Sarcomas Squamous cell carcinoma of temporal bone and external auditory canal Malignant peripheral nerve sheath tumours Thyroid malignancies Lymphoma Benign tumours like meningioma, osteoblastoma, osteochondroma	RT causes double-stranded brakes in DNA inducing mutations leading to malignant transformation Changes in microenvironment IMRT has 0.5% increased risk of malignancy compared to 3D conformal therapy, and double the risk compared to conventional RT, due to low dose scatter radiation delivered to non-target tissue prolonged beam-on time. Proton therapy is associated with a lower risk as compared to photon therapy	4-27 years

**Table 4.** Imaging features of post-radiotherapy (RT) complications.

Complications	Imaging features
Mucosal necrosis	The absence of mucosal enhancement on CT and MRI with/without ulceration Gas locules surrounding the lesion, better appreciated on CT suggestive of tissue necrosis. However, deep ulcers with associated solid enhancement should raise the suspicion of recurrence and clinical evaluation and close follow-up would help in confirming
Trismus	Pterygoid muscle volume loss with increased T2 signal and enhancement strictly within the confines of radiation field seen as a linear boundary
Osteoradionecrosis	Mandible most commonly affected (due to superficial location and poor vascularity). Can also be seen in skull base, temporal bones, maxilla, and hyoid. With parotid sparing techniques of IMRT, xerostomia has reduced and so has the incidence of periodontal disease which acts a risk factor for ORN. On CT, ORN appears as patchy lytic areas, disorganized sclerosis, cortical destruction, pathological fracture, loss of trabecular pattern, with or without fistulization to the skin The presence of cortical defects away from primary tumour site is highly suggestive of ORN. Secondary osteomyelitis and bone sequestration can also be seen. On MRI, a new abnormal marrow signal intensity (intermediate to low on T1 and intermediate to hyperintense on T2), cortical destruction without any soft tissue component, diffuse, intense enhancement of the abnormal marrow is suggestive of ORN
Chondroradionecrosis	Fragmented, collapsed thyroid cartilage with or without the presence of gas bubbles on CT. Effusion in cricoarytenoid joint leading to anterior dislocation of arytenoid cartilage, eventually increasing lysis leading to its disappearance. Sclerosis of the cricoid cartilage may be seen. Post-contrast enhancement and loss of high medullary signal on TWI within the ossified cartilage is suggestive of CRN on MRI
Vascular complications	Internal jugular vein thrombosis can occur, seen as filling defect on CECT or CEMRI. Mural thickening of carotid artery suggestive of atherosclerosis may be seen. Thrombus can be directly visualized on colour doppler. Carotid sparing IMRT have reduced the incidence of these vascular complications. Rare vascular complications include pseudoaneurysms and carotid arterial blowouts
Radiation induced lung damage	Radiation pneumonitis is seen as focal ground glass densities, with or without consolidation on CT. Radiation fibrosis manifests as a well demarcated area of volume loss, linear scarring, and traction bronchiectasis on CT
Neurological complications	Incidence of brain necrosis has significantly reduced due to newer RT techniques which contour out the brain from the radiation field. Radiation induced medial and inferior temporal lobe necrosis is seen as ring enhancing lesion with perilesional oedema on MRI. Cranial and brachial neuropathy manifest as thickening and enhancement of the affected nerves, and roots and trunks, respectively. Acute radiation cord injury is reversible but delayed post-RT cord injury appears as enlarged T2 hyperintense cord showing enhancement. Radiation induced HSE manifests usually as unilateral temporal lobe and insular cortex T2 and FLAIR hyperintensities showing diffusion restriction
Radiation induced neoplasm	It is defined as a tumour with a different histology occurring within an irradiated field at a site away from the original tumour after a latency period of 5 years or more. Imaging findings will depend upon the new malignancy that has developed

Abbreviations: CECT = contrast-enhanced CT; CEMRI = contrast-enhanced MRI; CRN = chondroradionecrosis; FLAIR = fluid attenuated inversion recovery; HSE = herpes simplex encephalitis; IMRT = intensity modulated radiotherapy; ORN = osteoradionecrosis; RT = radiotherapy.

**Figure 9.**
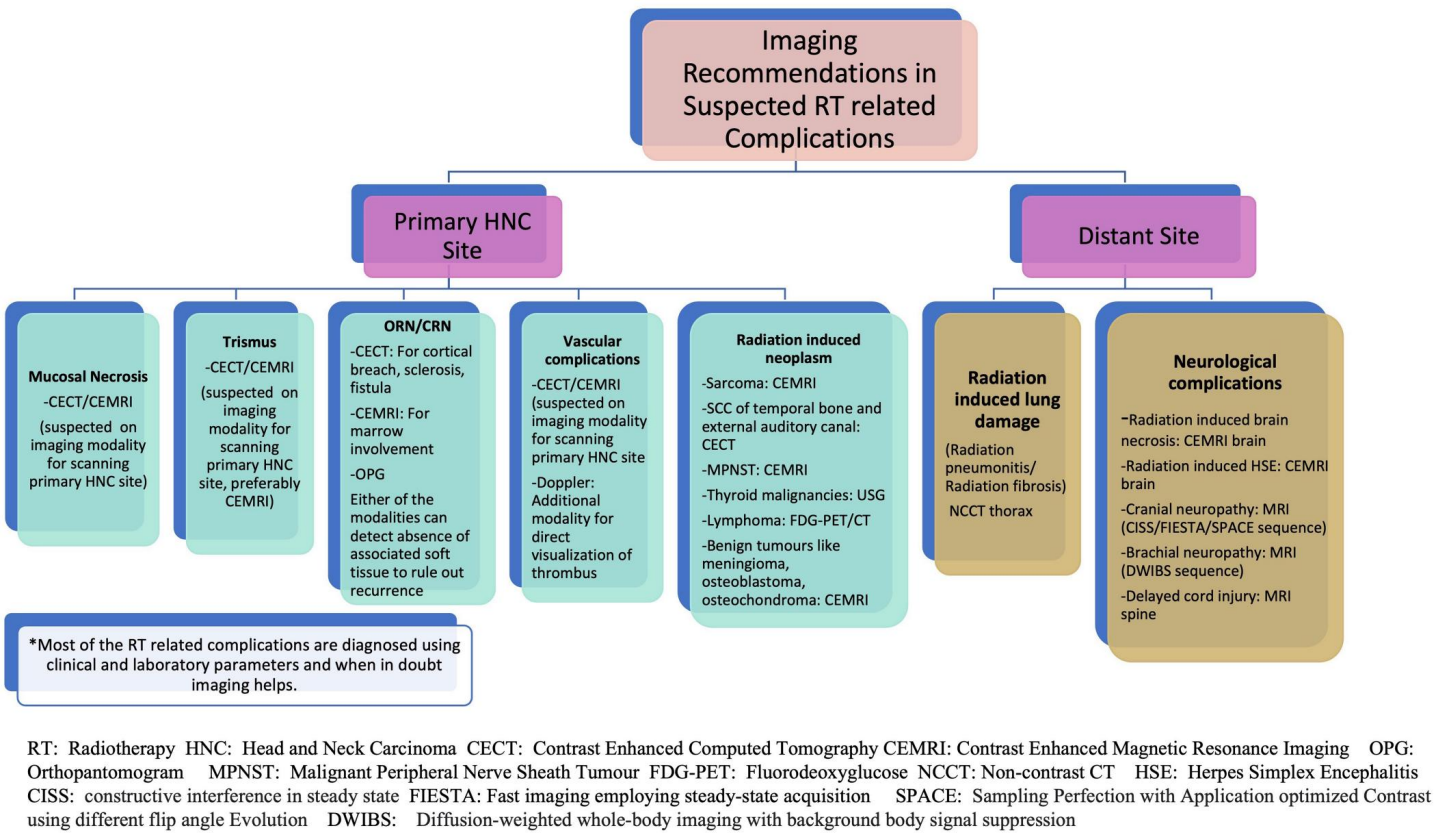
Flowchart on imaging recommendations for suspected RT related complications in head and neck carcinoma.

Flowchart in Figure 9 enumerates the imaging recommendations for suspected RT related complications.

### Induction chemotherapy/NACT

The main objectives of NACT in locally advanced HNSCC are organ preservation (laryngeal or hypopharyngeal squamous cell carcinomas), tumour shrinkage to render it resectable, reduce surgical margins, reduce distant metastasis, and reduce intensity of subsequent RT/CRT.^2^^,^^7–9^^,^^54^ Febrile neutropenia, diarrhoea, and mucositis are the common complications after induction chemotherapy, which are diagnosed clinically and do not require imaging.^7^

#### Response assessment after NACT on imaging

Responders to NACT tend to respond better to subsequent RT and have an improved overall survival (OS), hence, adequate response assessment on imaging is of paramount importance.^8^ On post-NACT imaging, if the tumour shrinks from sites which are critical for determining resectability, then tumour is considered to be operable and surgery is performed (with adjuvant CRT/RT).^9^^,^^10^ Conversely, if the tumour persistently or newly involves a critical structure after NACT on imaging, the tumour is inoperable. Those who are inoperable after NACT, undergo adjuvant CRT/RT. Though Response Evaluation Criteria In Solid Tumours (RECIST 1.1); which relies on single largest dimension for response assessment, is commonly used, it is inadequate for response evaluation of HNSCC.^8^^,^^10^ RECIST relies on size criteria for response assessment, however, operability of oral cavity tumours and neck dissection post-NACT is determined by shrinkage of tumour from certain sites; hyoid bone (provided tumour does not infiltrate hyoid bone), oedema extending till zygoma, high infratemporal fossa involvement, tumour showing <270° abutment with carotid vessels (for further reduction), in order to get adequate surgical margins, or from vallecula to get better visibility during surgery. Hence, if a tumour shows partial response using RECIST, but does not shrink from the above-mentioned sites, then the tumour still remains inoperable, therefore RECIST is inadequate for response assessment in such cases. So, in these cases, in addition to size criteria, indication for NACT should also be taken into consideration and resolution of tumour from the above-mentioned sites should be considered as a measure of adequate response post-NACT.

RECIST, however, is adequate for assessing response in laryngeal and hypopharyngeal tumours where reduction in size of tumour is the basic intention as further treatment plan is adjuvant RT and not surgery.

**Figure 10.**
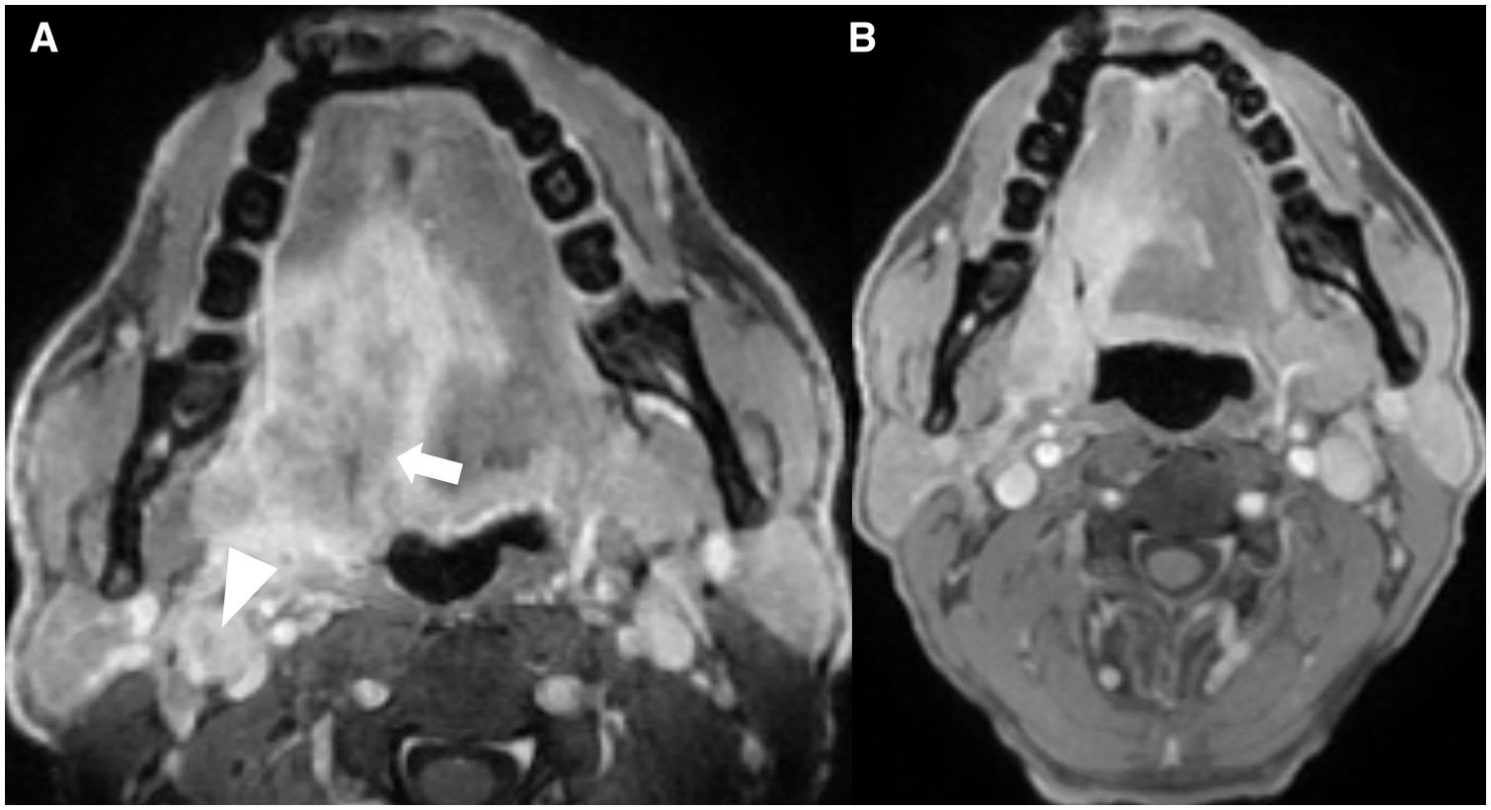
(A) Pre-neoadjuvant chemotherapy (NACT) MRI shows T4bN1 carcinoma right side base of tongue (arrow) with a metastatic right level II node (arrowhead). (B) Post-NACT MRI shows partial response of the primary as well as the node as per RECIST 1.1.

Prognostic value of change in tumour volume on post-NACT scan is yet to be explored, however, delineation of accurate tumour margin on post-NACT scan is challenging owing to associated treatment-related changes, hence, definite response assessment in such patients can be made on FDG-PET/CT scan done 12 weeks after adjuvant CRT/RT.^8^Figure 10 shows post-NACT response on MRI in carcinoma tongue.

### Immunotherapy

Metastatic and recurrent HNC have been the main target for immunotherapy benefitting 15%-20% of the patients.^14^ Various studies are ongoing to channelise the benefit of immunotherapy in neoadjuvant setting, as well as in combination with RT.^11^^,^^55–57^ Post-immunotherapy response is assessed based on single largest axial dimension using modified RECIST (iRECIST) which takes into account the phenomenon of pseudoprogression seen with immunotherapy.^58–60^ Common complications after immunotherapy are pneumonitis and colitis for which imaging might be required. Other post-immunotherapy complications like oral mucositis, autoimmune diabetes, and rash, are diagnosed clinically.^11^

### Post-treatment imaging in HNSCC: recurrence

Tumour recurrence manifests in one or more of the following ways:

#### Recurrence at the primary tumour site

After radical surgery with or without flap reconstructions, recurrence can manifest at the operated primary tumour bed, surgical or flap margins, or at the under surface of flap, as shown in Figure S2.^21–23^ Tumour recurrence deep to flap reconstructions evade detection on clinical examination, hence, the role of imaging in such cases. Earliest sign of recurrence on imaging may be induration of skin, subcutaneous tissue, or fatty component of flap, and progressive soft tissue thickening along with local infiltration.^23^ Tumour recurrence appears as enhancing infiltrating mass, mildly hyperdense on non-contrast CT scan and shows intermediate signal intensity on T2WI MRI, with or without bone destruction.^22^ Bone destruction is best visualized in the bone window setting on CT scan or on non-contrast T1WI.^23^ Any new onset nodularity or focal mucosal enhancement should raise the suspicion of recurrence. Oedema has attenuation less than muscle on CT, thus differentiating it from recurrence.^22^ Post-operative vascularized scar is seen as an enhancing ill-defined soft tissue on CECT and CEMRI, and is of T2 intermediate signal intensity similar to that of recurrence. However, on follow MRI, vascularized scar undergoes retraction and shows T2 hypointensity suggestive of fibrosis, thus, differentiating it from recurrence.^22^^,^^23^ Multiparametric MRI using diffusion weighted imaging (DWI) and apparent diffusion coefficient (ADC) can differentiate tumour recurrence from post-operative fibrosis and inflammation, as recurrence shows hyperintensity on DWI and signal drop on ADC (diffusion restriction), whereas low ADC value is not seen with post-operative changes.^22^ One of the studies have shown that an ADC value ≤1.43 × 10^−3^ mm^2^/s can be used for differentiating locoregional recurrence/residual tumour from post-treatment benign changes.^61^

After RT/CRT, focal heterogeneously enhancing infiltrative mass amidst the backdrop of homogeneously enhancing post-therapy fibrosis, should be viewed with suspicion for recurrence on CECT or CEMRI. The presence of enhancing soft tissue mass adjacent to ORN or solid enhancement adjacent to mucosal necrosis should raise the suspicion of recurrence on CECT or CEMRI and biopsy should be performed for confirmation.^22^^,^^38^ The absence of FDG uptake at the primary site after RT/CRT safely excludes residual disease when performed after 12 weeks.^39^

If the T2 signal intensity of the soft tissue in the post-treatment scan is of intermediate signal intensity (Figure S3) or similar to that of the tumour in the baseline/treatment naïve scan, then it is highly suggestive of recurrence. Table S2 shows imaging differentiation of recurrence from abscess, seroma, fibrosis, vascularized scar, oedema, and osteoradionecrosis.

#### Perineural recurrence

Recurrent tumour carries a higher risk of perineural spread, best detected on MRI along with its intracranial extension. Post-operative granulation tissue and scarring can sometimes pose difficulty in interpretation, for which correlation with clinical symptoms might be helpful, as unexplained symptoms of perineural spread appear prior to being evident on images, and follow-up imaging might be required in some cases.^22^^,^^62–64^ Perineural spread commonly occurs retrogradely towards the brainstem, may show skip lesions, and may further disseminate to leptomeninges, hence, MRI protocol for evaluating perineural recurrence should include entire course (extracranial and intracranial) of the involved nerve along with brain.^64^^,^^65^ Nerve thickening can occur on first follow-up scan after RT as part of post-treatment change if the nerve is within the field of RT, however, further increase in the nerve thickening with increase in enhancement, widening of skull base foramen, intermediate signal intensity on T2, and diffusion restriction, suggest perineural recurrence as shown in Figure S4.

#### Neck nodal recurrence

Oral cavity carcinomas usually metastasize to levels I, II, and III nodes, whereas oropharyngeal and supraglottic laryngeal carcinomas frequently metastasize to levels II, III, and IV nodes.^22^ Carcinomas involving nasopharynx, hypopharynx, and base of tongue usually show metastases to levels II, III, IV, and V nodes. Carcinomas of the nasopharynx, oropharynx, base of the tongue, and supraglottic larynx commonly show bilateral nodal metastases.^6^^,^^22^ On CECT/CEMRI, metastatic nodes show round shape, loss of fatty hilum, the presence of necrosis, heterogeneous enhancement, and irregular capsule.^62^ Mildly increased enhancement and diffusion restriction (high signal on DWI and signal drop on ADC) are the keys to identifying nodal recurrence amidst post-treatment changes.^22^ Ultrasonography may be performed followed by fine needle biopsy (FNB), if nodal involvement is indeterminate on cross sectional imaging. FDG-PET/CECT is recommended 10-12 weeks after RT or CRT in case of suspected recurrence or to assess neck response to RT.^2^^,^^39^^,^^66–68^ The absence of FDG uptake at the lymph nodal site after RT/CRT safely excludes residual disease when performed after 12 weeks.^39^ PET-CT guided active surveillance for locally advanced nodal metastasis (N2/N3) in HNSCC patients treated with radical CRT results in lower costs and complications.^69^

Patients with negative FDG-PET/CECT at first follow-up have better progression free survival (PFS) and OS.^70^^,^^71^ In follow-up of advanced nodal disease, if there is no FDG uptake or no enlarged node, then it suggests complete response and if intense FDG uptake is detected within normal sized or enlarged node, it is indicative of recurrence.^68^ Mild or no FDG uptake in enlarged node or mild FDG uptake in the normal sized node point towards equivocal findings and warrant ultrasonography guided FNB.^68^

#### Distant metastasis

FDG-PET/CECT or CECT thorax can detect lung metastasis and can pick up any synchronous malignancies in the upper aerodigestive tract or lungs, which are so common with HNSCC due to tobacco use and alcohol consumption.^2^^,^^22^^,^^38^ CEMRI is the modality of choice for detecting brain metastasis when patient has neurological symptoms.

### Recurrence versus second primary

Most of the researchers across the globe are in agreement that a malignant tumour arising from the same site or within 2 cm of the previous tumour, with a disease free interval (DFI) of less than 3 years, is a recurrent tumour. On the other hand, a malignant tumour arising from a separate head and neck cancer subsite, irrespective of the DFI, is a second primary tumour.^72^ Dilemma exists regarding categorization of a tumour into recurrent or second primary when the distance of malignant tumour from the primary site is more than 2 cm and/or the DFI is more than 3 years. Warren and Gates criteria are most widely followed, in which any malignant tumour arising from the same site, irrespective of its distance from the previous tumour, and irrespective of DFI, is a recurrent tumour and not a second primary.^73^

### Post-treatment imaging in HPV positive HNSCC

In head and neck region, HPV positive carcinomas are mostly oropharyngeal in origin.^6^ Only a few tumours of oral cavity, larynx, and paranasal sinuses are HPV related. As per the NCCN guidelines, treatment of HPV positive OPC includes surgical resection along with ipsilateral or contralateral nodal dissection, or definitive RT in the early stages, and CCRT or NACT followed by RT or systemic therapy/RT or surgical resection of the primary and nodal disease in the later stages.^6^ HPV positive OPC have a favourable prognosis with a longer asymptomatic post-treatment period, but with a similar rate of distant metastases as compared to HPV negative OPC (10%-15%).^74^ The pattern of recurrence of HPV positive OPC is however different with more distant and disseminated metastases in multiple atypical organs, unlike HPV negative OPC which present with local recurrence.^74^^,^^75^ One of the studies have shown that lungs is the commonest site of metastasis.^74^ If NACT is given in HPV positive HNSCC, then CECT/CEMRI can be used for response assessment depending upon the imaging modality used for pre-treatment evaluation. Otherwise, PET-CT is the modality of choice for post-treatment evaluation of HPV positive OPC to rule out local recurrence and distant metastasis after 16 weeks.^74^^,^^76^ One of the studies have shown that higher risk of distant metastases and lower locoregional failure can be predicted in HPV positive OPC using mid-treatment PET-CT by identifying volumetric nodal metabolic response.^76^^,^^77^ Most of the distant metastases occur 6 months after treatment completion, hence routine surveillance using FDG-PET/CT may be suggested in HPV positive OPC.^74^ HPV positive OPC usually presents at a younger age and has a better prognosis as compared to HPV negative OPC, hence they are predisposed to acute and long term toxicity from definitive RT/CRT.^78^ Dynamic Imaging Grade of Swallowing Toxicity (DIGEST) score using modified barium swallow imaged with videofluoroscopy can assess radiation induced swallowing inefficiency and dysphagia.^74^^,^^79^ Increase in mid-therapy T2 SI, decrease in T1SI, and increase in T1 post-contrast MRI throughout the therapy in pharyngeal constrictor muscles can serve as a biomarker for radiation induced dysphagia.

### Indications and recommendations on post-treatment imaging

NCCN and ESMO guidelines recommend CECT and/or CEMRI of the primary site within 3-4 months of surgery or primary treatment in patients with locoregionally advanced cancers to serve as a baseline scan prior to commencement of adjuvant therapy.^2^^,^^6^ Usually, review of the RT planning study is done by the radiologist to rule out any residual disease after surgical resection prior to commencing adjuvant RT, and in case of any concerns, a diagnostic imaging study is performed.

**Figure 11.**
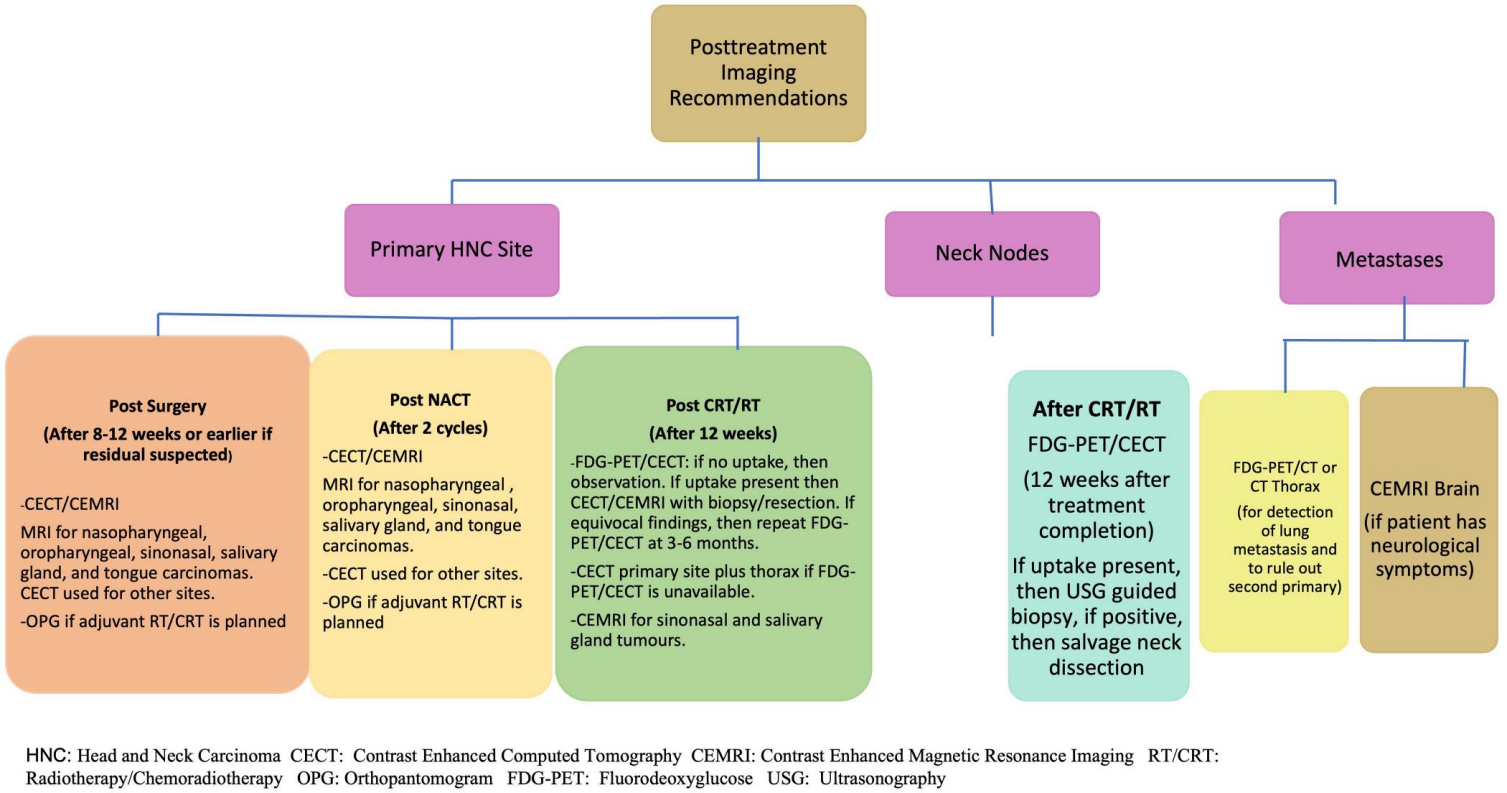
Flowchart on post-treatment imaging recommendations.

NCCN does not recommend routine imaging surveillance in an asymptomatic treated patient with negative examination if FDG-PET at 3 months has been negative, however, routine annual imaging may be performed for areas difficult to visualize on clinical examination.^6^ Flowchart on the site and treatment specific imaging modality to be used for post-treatment assessment of HNSCC based on NCCN and ESMO guidelines is shown in Figure 11. Both MRI and PET-CECT may be required for follow-up of patients with tumours near or involving the skull base, for evaluation of perineural, intracranial, or intraorbital tumour extension.

Suggested CT and MRI protocols for post-treatment assessment of HNSCC are provided in Table 5.^18^^,^^63^^,^^80^^,^^81^ For performing FDG-PET/CECT, 5 MBq/kg body weight is the usual dose of 18FDG and this is combined with CECT.^80^ Standard area of coverage with FDG-PET/CT is skull base to mid-thigh.^82^

**Table 5.** Suggested CECT and CEMRI protocol for post-treatment imaging.

Parameter	Description
CECT	
Scan extent	Base of skull till thoracic inlet
Slice thickness	0.75 mm
Intravenous contrast	80 mL of contrast at the rate of 3–5 mL/s should be administered and acquisition of images should be 20–25 s after contrast injection
Post-scanning reformats	Multiplanar reformats in all three planes; axial, sagittal, and coronal, in soft tissue window and bone algorithm reconstructions
CEMRI	
Scan extent	Base of skull till thoracic inlet
Slice thickness	3 mm section thickness and 1 mm gap
Intravenous contrast	0.1 mmol/kg Gadolinium should be given as a bolus at the rate of 2 mL/s
Sequences	T1WI, T2, STIR, and post-contrast T1WI in all three planes using 3DSPGR, DWI and apparent diffusion coefficient in axial plane (mainly for neck nodes) For perineural spread: Heavily T2 weighted gradient echo sequence; CISS/ FIESTA/SPACE (for intracranial extraaxial segment of nerve) 3D unenhanced MR neurography along with 3DT1W (0.5–0.8 mm) post-contrast for extracranial nerves

Abbreviations: 3DSPGR = 3D spoiled gradient recalled acquisition in steady state; CECT = contrast-enhanced CT; CEMRI = contrast-enhanced MRI; CISS = constructive interference in steady state; DWI = diffusion weighted imaging; FIESTA = fast imaging employing steady-state acquisition; SPACE = sampling perfection with application optimized contrast using different flip angle evolution; STIR = short tau inversion recovery; WI = weighted imaging.

Two consecutively negative FDG-PET/CECT performed within a six-month period has a negative predictive value of 98% and obviates the need for further imaging if clinical signs of recurrence are absent.^83^ Routine follow-up imaging is not recommended if FDG-PET/CECT done 12 weeks after treatment completion with CRT/RT is negative, unless there is a clinical suspicion of recurrence. Both MRI and PET-CECT should be used for follow-up after CRT and in a suspected case of recurrent nasopharyngeal carcinoma.^84^ When patient has been successfully treated with surgery without the need for adjuvant therapy (no high-risk features on post-operative specimen on histopathology), then no further follow-up imaging is required.^6^

Indications for multidisciplinary tumour board discussions after treatment completion are as follows:

- To confirm recurrence radiologically and pathologically.
- To decide upon feasibility of salvage surgery in case of local recurrence without distant metastasis.
- To decide upon salvage neck dissections in case of regional nodal residual/recurrent disease without distant metastasis.
- To decide upon palliative RT and/or immunotherapy for those with distant recurrent disease.
- To differentiate osteoradionecrosis from recurrence and decide upon further management plan.

### Assessment of post-treatment imaging findings: role of Hopkins NI-RADS

#### Hopkins

Hopkins is a five-point qualitative post-therapy FDG-PET/CECT based response assessment criteria for HNSCC, having excellent negative predictive value and distinguished role in predicting PFS and OS.^85^^,^^86^Table S3 shows the Hopkins criteria for post-therapy assessment.^86^

#### Neck imaging reporting and data systems

**Figure 12.**
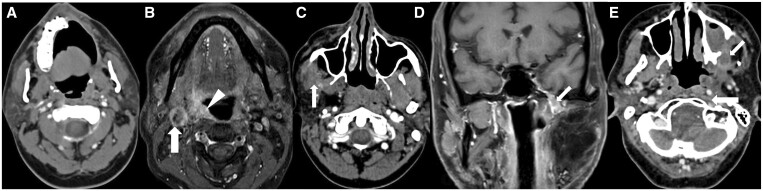
(A) Left infrastructure maxillectomy case of carcinoma left upper alveolus shows expected post-operative changes on CECT (NI-RADS 1). (B) CEMRI in a post-tonsillectomy case of carcinoma right tonsil shows ill-defined heterogeneously enhancing diffuse mucosal thickening in the right tonsillar fossa region (arrowhead), involving the tonsilolingual sulcus with extension into parapharyngeal space which has low suspicion for recurrent disease (NI-RADS 2a at primary site). In addition, a metastatic necrotic right level II node (arrow) is seen (NI-RADS 4 for neck node). (C) CECT of an operated case of carcinoma right buccal mucosa post adjuvant chemoradiotherapy shows mildly heterogeneously enhancing diffuse soft tissue (arrow) along superior aspect of post-operative scar abutting temporalis muscle suggestive of low suspicion for recurrence (NI-RADS 2b). (D) Post-operative case of carcinoma left buccal mucosal shows a peripherally enhancing central hypodense lesion communicating with left maxillary sinus thorough the eroded posterolateral wall which has high suspicion for recurrence (NI-RADS 3). (E) CEMRI in an operated case of left buccal mucosa carcinoma with flap shows definitive recurrence in the form of perineural spread (arrow) at the flap margin reaching up to left foramen ovale (NI-RADS 4).

American College of Radiology (ACR) developed NI-RADS for comprehensive post-treatment HNSCC and non-squamous HNC (tumours of salivary gland, nasal cavity and paranasal sinuses, orbit and thyroid gland) evaluation and management which includes a lexicon to differentiate benign from malignant post-treatment findings, reporting templates with defined level of suspicion and management recommendations, and post-treatment surveillance imaging, which can be used with CECT with or without FDG-PET and also with MRI.^81^^,^^87^^,^^88^ NI-RADS includes categories 0-4 (incomplete to definite recurrence) on FDG-PET/CECT and MRI, with separate descriptions for primary site and neck in each of them, with their management implications. NI-RADS should be followed while recording imaging findings after definitive treatment completion, and not while the patient is on treatment. Table S4 shows NI-RADS categories on FDG-PET/CECT for primary site and neck node.^87^^,^^88^ MRI NI-RADS categories for primary tumour are shown in Table S5. CT and MRI of treated HNSCC cases along with their NI-RADS categories are shown in Figure 12 and Figures S5-S9.

Post-treatment surveillance recommendations as per the ACR NI-RADS committee suggests PET-CECT to be performed at 8-12 weeks after completion of definitive treatment and if negative, CECT or PET-CECT to be performed 6 months later. If the first CECT is negative, then only CECT neck should be performed 6 months later and if this second CECT is negative, then CECT neck and chest 12 months later should be done. If two consecutive PET-CECTs are negative, then there is no need for further surveillance imaging.^87^

### Advanced imaging techniques for post-treatment HNSCC evaluation

Intravoxel incoherent motion (IVIM), dynamic contrast-enhanced MRI (DCE-MRI), and blood oxygen level dependent (BOLD) MRI are the functional MRI techniques being explored for their role in post-treatment HNSCC evaluation, in addition to DWI.

IVIM non-invasively assesses perfusion and generates quantitative map of small functional blood vessel density, which is a marker of angiogenesis (known to play a role in tumour growth).^89–92^ IVIM calculates parameters like pure diffusion coefficient (*D*), microvascular volume fraction (*f*), and perfusion-related incoherent microcirculation (*D*^∗^), thus segregating diffusion from perfusion.^92^ Low pre-treatment *D* and *f* values and increase in *D* during treatment entail a favourable response to treatment in HNSCC.^93^ Differentiation of malignant tumour from CRT related fibrosis was possible using *D* value with a sensitivity and specificity of 100% in one of the studies.^93^^,^^94^ One of the studies has shown significant difference in pre-CRT *D* value between responders and non-responders using IVIM.^95^ In addition, IVIM curtails the cost of contrast agents, acquisition times, and is a boon to those in whom contrast agents are contraindicated. However, IVIM technique, acquisition parameters including *b*-values, and algorithms for quantitative image analysis need to be standardized.^91^

DCE-MRI uses gadolinium contrast to image tissue perfusion. One of the most common parameters derived from DCE-MRI is *K*^trans^, which has shown a strong predictive association with progression free and overall survival in advanced HNSCC.^96–98^*K*^trans^ is a volume constant which provides information about the tumour perfusion and vascular permeability. Higher pre-treatment *K*^trans^; mean ± SD *K*^trans^ of 0.90 ± 0.54 min^−1^ and median of 0.88 min^−1^, from metastatic nodes, has shown to have a better 5-year overall survival in HNSCC as compared to those with a low pre-treatment *K*^trans^.^97^

DCE-MRI has the potential to differentiate between local recurrence and post-treatment change with the help of time-signal intensity (TSI) curves. Plateau or wash-out (type II or III) TSI curves are seen in local recurrence due to recurrent or residual tumour showing early and intense enhancement owing to leaky vessels, whereas, progressive increment (type I) TSI curves are seen in post-treatment change predominantly owing to persistent delayed enhancement in scar, as shown in one of the studies.^99^ DCE-MRI can also predict ORN by demonstrating increased DCE parameters in ORN affected mandible as compared to normal mandible.^74^^,^^100^

BOLD MRI is a non-invasive technique to evaluate tumour hypoxia, an entity known to reduce effectiveness of CRT and associated with unfavourable outcome in HNSCC.^101^

Paramagnetic effect of blood deoxyhaemoglobin reduces the signal intensity on T2* images and this forms the basis of identifying tumour hypoxia on BOLD imaging. When oxygen or carbogen is inhaled, there is increase in diamagnetic oxyhaemoglobin which results in increased T2* signal intensity within tumour and the difference in tumour oxygenation is detected by BOLD, which is then used to select patients suitable for anti-hypoxia treatment in radiotherapy.^101^^,^^102^

Fluoro-misonidazole (^18^F-MISO) and ^18^F-fluoroazomycin-arabinofuranoside (^18^F-FAZA) are the two radiotracers that can be combined with PET to evaluate tumour hypoxia.^96^ One of the studies has shown that tumour hypoxia quantification using the FMISO-PET parameters such as peak tumour-to-background-ratio (TBRpeak); calculated as the standardized uptake value (SUV) at peak divided by the mean SUV within the background volume of interest, with a cut-off 2.0 (*P* < .001) in week 2, and residual hypoxic volume with 1.6-fold muscle SUV _mean_ (rHV _1.6_) cut-off 0.2, (*P* < .001), can enable stratification of patients into low (those with a lower than cut-off values indicating lesser tumour hypoxia) and high (those with a higher than cut-off values indicating more tumour hypoxia) risk of resistance to treatment and thus locoregional recurrence.^103^ All the voxels within SUV uptake of FAZA for the gross tumour volume GTV_FAZA-T_ demonstrating a tumour-to-muscle value equal to or above 1.4 was defined as the hypoxic volume by FAZA PET/CT in one of the studies, and it correlated with poor outcomes in post-RT HNSCC patients.^104^

### Systematic review of multimodality post-treatment HNSCC studies

Table S6 shows systematic review of multimodality post-treatment HNSCC studies in the last 15 years.^105–109^ Pubmed search was conducted for major imaging based post-treatment HNSCC studies in the last 15 years in adult patients >19 years of age reported in English language. Only comparative studies having sensitivity and specificity data were included in the table and those involving single imaging modality were discarded.

## Conclusion

Given the shift towards multimodal treatment for head and neck cancers, interpreting post-treatment scan has become complex. Radiologists must possess comprehensive understanding of different surgical techniques and radiation therapy effects to accurately diagnose and manage recurrence and complications. While imaging guidelines vary globally, NCCN recommendations are widely followed.

For locally advanced HNSCC, depending on the specific subsite of the carcinoma, either CEMRI or CECT should be conducted within 3-4 months following surgical resection. This aims to exclude any residual disease/early recurrence before initiating adjuvant CRT/RT. If such a diagnostic scan has not been performed, then typically, the radiologist should review the radiotherapy planning study to ensure the absence of residual disease/early recurrence, and if any concerns arise, further diagnostic imaging studies should be undertaken.

After CRT/RT, it is recommended to utilize FDG-PET/CECT for assessing response 12 weeks post-treatment. If the results are negative, routine imaging surveillance is unnecessary unless recurrence is suspected. In cases where recurrent nasopharyngeal carcinoma is suspected, both MRI and PET-CECT should be employed for follow-up evaluation.

In the event of intense FDG uptake detected within a normal sized or enlarged node on follow-up PET-CECT, an ultrasound-guided biopsy is recommended to confirm recurrence.

If a patient has undergone successful surgery without the necessity for adjuvant therapy, meaning there are no high-risk features on the post-operative histopathology specimen, further follow-up imaging is unnecessary.

Annual imaging on a routine basis may be considered for regions that are challenging to visualize during clinical examination.

Following completion of treatment, it is customary to conduct multidisciplinary tumour board discussions. These discussions serve several purposes, including confirming recurrence, determining the necessity for salvage surgeries and neck dissections in instances of local recurrence or residual/regrown regional disease without distant metastasis, devising palliative therapies for distant recurrence, and distinguishing between osteoradionecrosis and recurrence to strategize further management.

In HPV positive oropharyngeal squamous cell carcinoma, routine surveillance with FDG-PET/CT may be contemplated. This is because the majority of distant metastases tend to manifest within 6 months following completion of treatment.

NI-RADS guidelines should be adhered to for documenting post-treatment imaging findings after completion of treatment for both squamous cell carcinomas of the head and neck and non-squamous cell cancers.

There is a necessity to prospectively validate the efficacy of advanced imaging techniques such as functional MRI, IVIM, and BOLD imaging. This validation should involve a larger sample size to ascertain their potential utility for predicting response to chemoradiotherapy.

## Supplementary Material

tqae207_Supplementary_Data
