# Supplementary material for: Comprehensive review of post-treatment imaging in head and neck cancers: from expected to unexpected and beyond
Source: Br J Radiol. 2024 Oct 11;97(1164):1898–914. doi: 10.1093/bjr/tqae207 (PMC11573130; doi:10.1093/bjr/tqae207)
Supplement: tqae207_Supplementary_Data [file tqae207_supplementary_data.zip › tqae207_Supplementary_Data/Supplement Tables 12.05.24.docx]

**Supplementary table 1: Head and neck surgeries and their pitfalls**

| **Type of head and neck surgery** | **Pitfalls/Complications** |
| --- | --- |
| Any type of head and neck surgery | Seroma  Infection  Abscess  Haematoma |
| *Minimally invasive surgeries*  Transoral robotic surgery (TORS)  Transoral laser microsurgery  (TLM) | Bleeding  Aspiration pneumonia  Oedema/stenosis  Subcutaneous emphysema  Local site infection  Cutaneous fistula |
| Free fibula flap mandibular reconstructions | *Early complications (< 6 weeks)*  Arterial insufficiency  Venous thrombosis  Skin island necrosis  *Late complications (> 6 weeks)*  Plate fracture  Wound dehiscence |
| Surgeries for oral cavity | Oroantral fistula  Orocutaneous fistula |
| Total laryngectomy | Pharyngocutaneous fistula |
| Free flap related complications | Delayed healing of flap donor site wound  Necrosis of native facial or neck skin  Partial flap necrosis  Total flap necrosis (flap failure)  Kinking and compression of the vascular pedicle  Wound infection  Salivary fistula  Neck haematoma |
| Pedicled flap related complications | Surgical site infection  Wound dehiscence  Flap failure  High risk for deep venous thrombosis  Pneand pneumonia |
| Neck dissection | Dehiscence  Chylous leak  Nerve injury (accessory nerve, marginal mandibular nerve, hypoglossal nerve, phrenic nerve, sympathetic trunk) |

**Supplementary table 2: Imaging differentiation of recurrence from abscess, seroma, fibrosis, vascularized scar, oedema and osteoradionecrosis.**

| **Pathology** | **Imaging appearance** |
| --- | --- |
| Recurrence | Focal nodular soft tissue  Mildly hyperdense on non-contrast CT scan  Heterogeneous contrast enhancement on CT and MRI  Intermediate T2 signal intensity or T2 signal intensity similar to the baseline/treatment naïve tumour.  Presence of diffusion restriction |
| Abscess | Hypodense on CT  Rim enhancement on CECT and CEMRI  T2 hyperintense  Presence of diffusion restriction |
| Seroma | Hypodense on CT  T2 hyperintense  No enhancement on CECT and CEMRI  Absence of diffusion restriction |
| Fibrosis | Isodense to muscle on non-contrast CT scan  No enhancement or delayed homogeneous enhancement  T2 hypointense |
| Vascularized scar | Ill-defined enhancing soft tissue on CECT and CEMRI  T2 intermediate signal intensity  Follow up MRI shows retraction and T2 hypointensity suggestive of fibrosis. |
| Oedema | Hypodense to muscle on non-contrast CT scan  T2 hyperintense  No diffusion restriction |
| Osteoradionecrosis | Affected bone shows patchy lytic areas, disorganized sclerosis, cortical destruction, pathological fracture, loss of trabecular pattern, with or without fistulization to the skin, on CT scan.  Absence of focal enhancing soft tissue  New abnormal marrow signal intensity (intermediate to low on T1 and intermediate to hyperintense on T2), cortical destruction without any soft tissue component, and diffuse, intense enhancement of the abnormal marrow on MRI. |

**Supplementary table 3: Hopkins criteria for post therapy FDG-PET/CECT based** **assessment for HNSCC.**

| **Score** | **Pattern of FDG uptake** | **Response category** |
| --- | --- | --- |
| 1 | FDG uptake at primary site and nodes less than IJV | Complete metabolic response |
| 2 | Focal FDG uptake at primary site and nodes more than IJV but less than liver | Likely complete metabolic response |
| 3 | Diffuse FDG uptake at primary site or nodes more than IJV or liver | Likely postradiation inflammation |
| 4 | Focal FDG uptake at primary site or nodes more than liver | Likely residual tumour |
| 5 | Focal and intense FDG uptake at primary site or nodes more than liver | Residual tumour |
| FDG-PET/CECT: Fluorodeoxyglucose-positron emission tomography/ contrast enhanced computed tomography, HNSCC : Head and neck squamous cell carcinoma, IJV: Internal jugular vein | | |

**Supplementary table 4:** **Neck Imaging Reporting and Data System (NI-RADS) for primary site and neck node on FDG-PET/CECT. [Adapted from American College of Radiology]**

| **For primary site** | | | |
| --- | --- | --- | --- |
| **NI-RADS category** | **Descriptor** | **Imaging findings** | **Recommendations** |
| 0 | Incomplete | No prior imaging available, new baseline study. | Score to be assigned after availability of prior imaging. |
| 1 | No evidence of recurrence | -Expected posttreatment changes.  -Soft tissue distortion without any discrete mass.  -Low density post radiation submucosal edema.  -No abnormal fluorodeoxyglucose (FDG) uptake.  -Diffuse linear mucosal enhancement due to benign radiation mucositis. | Routine surveillance |
| 2a | Low suspicion | -Focal non mass-like mucosal enhancement.  -Focal mild to moderate mucosal FDG uptake. | Direct visual inspection |
| 2b | Low suspicion | -Deep ill-defined soft tissue.  -No or little differential enhancement.  - Mild or moderate FDG uptake. | Short interval follow up (3 months), repeat positron emission tomography (PET). |
| 3 | High suspicion | -New or enlarging primary mass.  -Discrete nodule or mass with differential enhancement.  -Intense focal FDG uptake. | Biopsy (with or without image guidance) |
| 4 | Definitive recurrence | Pathologic proven recurrence or definite radiologic and clinical progression. | Clinical management |
| **For neck node** | | | |
| 1 | No evidence of recurrence | -No new node/abnormal node.  -Residual nodal tissue without any FDG uptake. | Routine surveillance |
| 2 | Low suspicion | - Residual nodal tissue without new necrosis or extranodal extension (ENE), showing mild/moderate FDG uptake.  -Newly enlarging node on CECT without uptake on FDG-PET.  -Discordance between PET & CECT/MRI. | Short interval follow up (3 months) |
| 3 | High suspicion | - Residual lymph nodal tissue showing intense FDG uptake.  -Growing lymph nodal tissue showing intense FDG uptake.  -New necrosis or ENE. | Biopsy |
| 4 | Definitive recurrence | Pathologic proven recurrence or definite radiologic and clinical progression. | Treatment of disease with or without biopsy. |

**Supplementary table 5:** **Neck Imaging Reporting and Data System (NI-RADS) for primary site on MRI. [Adapted from American College of Radiology]**

| **NI-RADS Score** | **NI-RADS Category** | **MRI findings** | **Recommendations** |
| --- | --- | --- | --- |
| 1 | No evidence of recurrence | Expected posttreatment changes.  Diffuse thin linear mucosal enhancement or submucosal oedema    No new focal nodular or mass-like soft tissue    Stable or reduced enhancement at skull base foramina and in perineural locations | Routine surveillance |
| 1f | No evidence of recurrence | *First post-treatment baseline scan:*    Resolution of tumour on pre-treatment study.    No focal nodular or mass-like soft tissue.    Reduced soft tissue and/or enhancement in skull base foramina and perineural regions. | Routine surveillance |
| 2a | Low suspicion of recurrence | Focal non-mass-like mucosal enhancement, superficial skin changes    Focal restricted diffusion | Direct visual inspection |
| 2b | Low suspicion of recurrence | Deep, ill-defined non-nodular soft tissue.    Soft tissue having different DWI, enhancement, or T1 and T2 signal characteristics as compared to the original tumour.  Intermediate T2 signal and intermediate enhancement of the soft tissue. | Short interval follow-up MRI (3 months) or PET to assess deep submucosal abnormality |
| 2f | Low suspicion of recurrence | *First post-treatment baseline scan:*    Partial resolution of tumour in comparison to pre-treatment study.    Same signal and enhancement of soft tissue in skull base foramina and perineural regions when compared to pre-treatment study.    New thin smooth enhancement in skull base foramina and perineural regions within radiation field. | For assessment of deep submucosal abnormality: Short interval follow-up MRI (3 months) or PET.      For assessment of perineural disease: Short interval follow-up MRI (3 months) |
| 3 | High suspicion of recurrence | New/enlarging nodule or mass at the primary site and signal characteristics and enhancement similar to original tumour.  Intense focal FDG uptake.    Increased soft tissue and/or enhancement in skull base foramina and perineural regions, and/or increased enhancement and nodular soft tissue along major nerves supplying the site of primary disease. | Biopsy |
| 4 | Definitive Recurrence | Definite radiologic and clinical progression or pathologically proven. | Clinical management, no biopsy required |

**Supplementary table 6: Systematic review of multimodality posttreatment Head and Neck Squamous Cell Carcinoma (HNSCC) studies in last 15 years^a^**

| **Study details** | **Imaging modality** | **Diagnostic performance** | | | | | **Key inference** |
| --- | --- | --- | --- | --- | --- | --- | --- |
|  |  | **S**  **(%)** | **Sp**  **(%)** | **PPV**  **(%)** | **NPV**  **(%)** | **A** |  |
| ***1. Semih et al***  ***2021 [107]***  Nasopharyngeal Carcinoma  Rx with RT  (n = 110) | MRI | 100 | 15.6 | 22.9 | 100 | 32.5 | FDG PET/CT is more effective than MRI for relapse detection at the primary tumor site in patients with NPC.  - Biopsy should be performed with SUV_max_ values more than 4.58 and avoided with lower values. |
|  | FDG-PET/CT | 75 | 71.9 | 40 | 92 | 72.5 |  |
| ***2. Pellini et al 2014 [108]***  Advanced oropharyngeal squamous cell carcinoma  Rx with CRT  (n = 36) | USG | 77.8 | 78.9 | 77.8 | 78.9 | 78.4 | -USG combined with FDG-PET/CT, had highest NPV (93.3%) for detecting residual nodal disease after CRT  -Neck dissection (ND) not required in patients with complete nodal response to CRT on combined USG and FDG-PET/  CT.  - Selective ND to be performed on detection of residual  disease in the neck. |
|  | MRI | 66.7 | 68.4 | 66.7 | 68.4 | 67.6 |  |
|  | FDG-PET/CT | 44.4 | 94.7 | 88.9 | 64.3 | 70.3 |  |
| ***3. Becker et al***  ***2018 [109]***  All HNSCC subsites Rx with RT/CRT  (n= 74) | Hybrid FDG-PET/DWIMRI | 97.4 | 91.7 | 92.5 | 97.1 | - | Hybrid FDG-PET/DWIMRI is excellent for detection and T classification of HNSCC after RT/CRT |
| ***4. Jajodia et al 2019 [110]***  All HNSCC subsites Rx with  Surgery/RT/CRT  (n = 62) | DWI | - | - | 96 | 83.3 | 96.6 | There was a statistically significant difference  in the ADC values between residual/recurrent disease (1.008 ± 0.220 × 10^-3^ mm^2^/s )  and post treatment  changes (1.69 ± 0.40 × 10^-3^ mm^2^/s) |
|  | Combined DWI and threshold ADC value of  1.3x10^−3^mm^2^/s | 94 | 83.3 | 95.9 | 83.3 | 93.6 |  |
| ***5. Ghanooni et al 2011 [111]***  Oral cavity, oropharynx, hypopharynx, and laryngeal carcinoma Rx with Surgery/RT/CRT  (n = 32) | MRI  Diagnostic performance at 4 months  Diagnostic performance at 12 months | 70  75 | 74  85 | 43  25 | 90  98 | -  - | -FDG PET/CT can distinguish between residual tumour and post RT changes, as early as 2 weeks after treatment of a primary HNSCC.  -FDG PET/CT and MRI have similar diagnostic performance for follow up, but sensitivity of FDG PET/CT at 4 months is higher. |
|  | FDG-PET/CT (non-contrast)  Diagnostic performance at 4 months  (SUV assessment)  Diagnostic performance at 12 months  (SUV assessment)  Diagnostic performance at 2 weeks post RT  (SUV cut off 5.8) | 92  100  86 | 87  86  85 | 64  27  50 | 98  100  97 | -  -  - |  |
| ^a^ Rx: Treatment, RT: Radiotherapy, CRT: Chemoradiotherapy, MRI: Magnetic resonance imaging, USG: Ultrasonography, FDG-PET/CT: Fluorodeoxyglucose positron emission tomography computed tomography, SUV: Standardized uptake value, ADC: Apparent diffusion coefficient, S: Sensitivity, Sp: Specificity, PPV: Positive predictive value, NPV: Negative predictive value, A: Accuracy | | | | | | | |
