# Supplementary figures and images for: Comprehensive review of post-treatment imaging in head and neck cancers: from expected to unexpected and beyond
Source: Br J Radiol. 2024 Oct 11;97(1164):1898–914. doi: 10.1093/bjr/tqae207 (PMC11573130; doi:10.1093/bjr/tqae207)

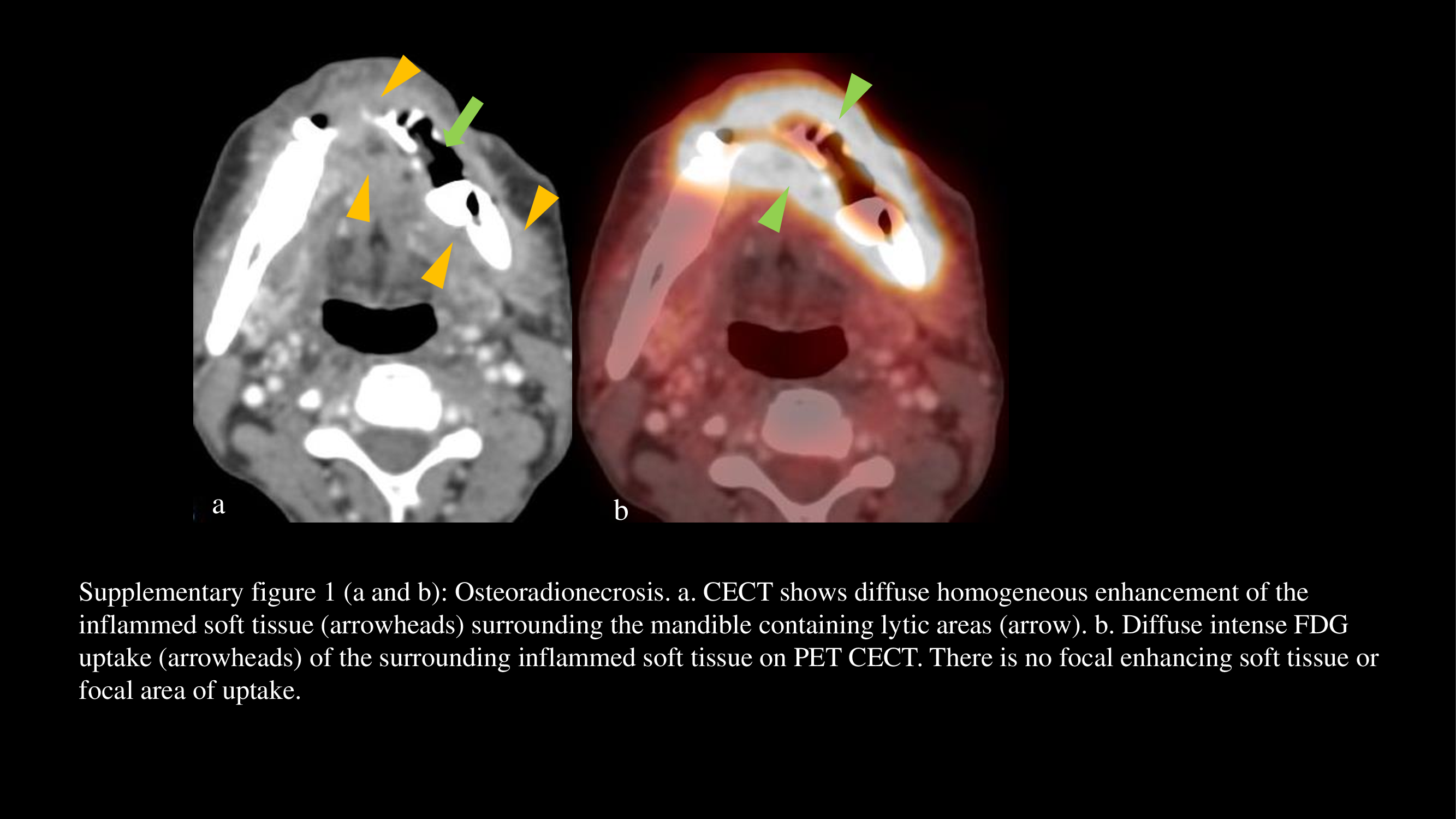

Supplement: tqae207_Supplementary_Data [file tqae207_supplementary_data.zip › tqae207_Supplementary_Data/Supplementary figures 12.05.24.tiff]
